# Resonant laser ionization and mass separation of ^225^Ac

**DOI:** 10.1038/s41598-023-28299-4

**Published:** 2023-01-24

**Authors:** Jake D. Johnson, Michael Heines, Frank Bruchertseifer, Eric Chevallay, Thomas E. Cocolios, Kristof Dockx, Charlotte Duchemin, Stephan Heinitz, Reinhard Heinke, Sophie Hurier, Laura Lambert, Benji Leenders, Hanna Skliarova, Thierry Stora, Wiktoria Wojtaczka

**Affiliations:** 1 KU Leuven, IKS, 3000 Leuven, Belgium; 2 JRC, Karlsruhe, Germany; 3 CERN, 1211 Geneva, Switzerland; 4 Belgian Nuclear Research Centre SCK CEN, Mol, Belgium; 5 Universiteit Gent, Gent, Belgium

**Keywords:** Experimental nuclear physics, Targeted therapies

## Abstract

$^{225}$Ac is a radio-isotope that can be linked to biological vector molecules to treat certain distributed cancers using targeted alpha therapy. However, developing $^{225}$Ac-labelled radiopharmaceuticals remains a challenge due to the supply shortage of pure $^{225}$Ac itself. Several techniques to obtain pure $^{225}$Ac are being investigated, amongst which is the high-energy proton spallation of thorium or uranium combined with resonant laser ionization and mass separation. As a proof-of-principle, we perform off-line resonant ionization mass spectrometry on two samples of $^{225}$Ac, each with a known activity, in different chemical environments. We report overall operational collection efficiencies of 10.1(2)% and 9.9(8)% for the cases in which the $^{225}$Ac was deposited on a rhenium surface and a ThO$_{2}$ mimic target matrix respectively. The bottleneck of the technique was the laser ionization efficiency, which was deduced to be 15.1(6)%.

## Introduction

Around 100 years ago, clinical oncologists were already aware that “cases of advanced cancer should receive roentgen therapy”^1^. This was in spite of the fact that very little information was available on the biological effects of ionizing radiation. Since then, the irradiation of tumors has become one of the primary cancer treatment modalities, with 50% of all patients worldwide being prescribed radiation therapy^2^. However, radiation therapy is only appropriate for direct treatment of localized tumors and cannot be employed if a tumor metastasizes^3^. Metastasis is the process by which malignant cells colonize a distant site within the body by spreading through the lymphatic or vascular system, and is often considered a detrimental stage in cancer development^4,5^. Once metastatic spread has begun, targeted radionuclide therapy (TRNT) could be considered as a viable treatment option for targeting even very small clusters of metastasized cancer cells with ionizing radiation^6,7^.

**Figure 1:**
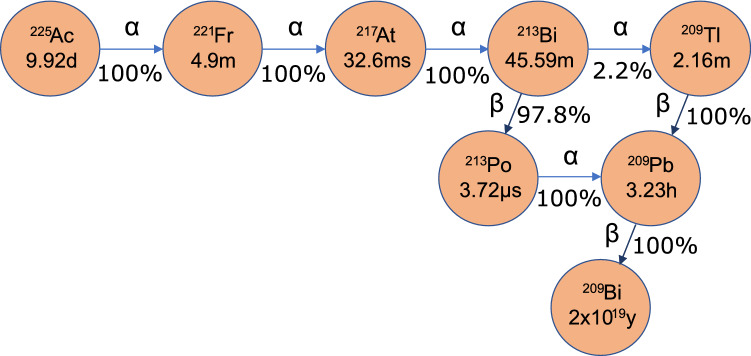
$^{225}$Ac  decay scheme. Half-lives of the isotopes are indicated in the circles, and decay modes and branching ratios are indicated along the arrows.

In TRNT, a biological vector molecule that targets tumor-specific antigens is labelled with an appropriate radionuclide and injected into a patient. An appropriate radionuclide is one whose half-life is matched to the pharmacokinetics of the targeting molecule. In this way, the decay probability of the radionuclide during the radiopharmaceutical circulation time is low. Once the radio-labeled vector binds to the tumor-specific antigen, the radionuclide remains within the malignant cell vicinity until it decays. The emitted radiation damages cancer cells predominantly through DNA lesions, but also by creation of free radicals in cytoplasm, local propagation of apoptosis signalling, as well as various immune response pathways^8–13^. In most instances of TRNT, beta radiation is used, with a path length of a few millimetres in human tissue. However, in the cases where the metastases consist of only a few cells, radio-isotopes that emit alpha radiation are desirable due to their range of a few human cell-lengths and their high linear energy transfer. Out of over 100 alpha-emitting nuclei, only a handful are suitable for TRNT. They must have a half-life on the scale of hours to days, form chemically stable complexes with appropriate bifunctional chelators to link to targeting molecules and have no toxic decay daughters. An overview of candidate radio-isotopes satisfying these prerequisites is given by Radchenko et al.^14^. Of these, one of the most promising is $^{225}$Ac. Its 9.92-day half-life^15,16^ is compatible with the circulation time of effective targeting molecules, and four high energy alpha particles are emitted in short succession in its decay chain, as shown in Fig. 1. There have been multiple in-vitro and in-vivo trials performed with $^{225}$Ac, with promising clinical results, a review of which is given by Morgenstern et al.^17^.

Despite encouraging results, one of the main challenges of further developing this technique remains the supply of the pure clinical-grade $^{225}$Ac  itself. Almost all of that currently used is eluted from three $^{229}$Th generators in Europe, the USA and Russia. Smaller capacity $^{229}$Th generators also exist but mostly for research rather than production purposes^18,19^. While the available amount of $^{225}$Ac  that can be obtained from these stocks could be increased due to increased access to the U.S. stock granted to TerraPower Inc.^20^, this reliance on generators is not viable to meet future demand^21^. Because of this, several alternative production pathways have been proposed. An overview of the potentials of such methods is given by Robertson et al.^22^ One of the main alternative methods for producing $^{225}$Ac  is the high-energy (>70 MeV)^23^ proton spallation of Th or U-based materials. While investigations into the cross section and production yields for $^{225}$Ac  produced in Th are ongoing, some measurements have been performed. Based on these values, it is suggested that >10 GBq could be produced in-target annually at isotope production facilities such as RIAR (Russia), LANSCE (USA), ARIEL (Canada) and MEDICIS (Europe)^23–26^. The U.S. DOE-funded ‘Tri-lab effort’ is also focused on increasing availability of accelerator-produced $^{225}$Ac  and establishing practices to be submitted in a drug master file^27^. For the accelerator-produced $^{225}$Ac  to be of clinical use, it must then be extracted from the irradiated target with high purity and efficiency. A common technique used for this is radio-chemical separation, with recent results indicating efficiencies of 85–98%^28–30^. A limitation of this technique is its inability to be isotopically selective. As a result, $^{227}$Ac  (t$_{1/2}$ = 21.8 years) contaminates the recovered $^{225}$Ac  with an end of collection activity fraction of 0.1-0.15%. This level of activity could lead to issues with radioactive waste handling for hospitals^21^, where in Europe the clearance level is 10 Bq/kg^31^ and the exemption level is 1 kBq^32^. Furthermore, pre-clinical research laboratories may also be adverse to handling substances with potential for long-lived $\alpha$-emitter contamination. Another approach that has been employed at TRIUMF is the elution of $^{225}$Ac  from $^{225}$Ra  that has been chemically separated from the target using cation exchange and separation chromatography. Here, only $^{223-226}$Ra remain in the target after a cool-down time of the order of a day, during which the other Ra isotopes decay. These Ra isotopes are then collected as eluates from extraction chromotography. Of these, $^{225}$Ra is the only isotope that decays via beta emission to form Ac. Thus by using the recovered Ra fraction as a generator, from which Ac is eluted, the recovered sample should consist almost entirely of $^{225}$Ac. The result is that the $^{227}$Ac  activity fraction of the resulting $^{225}$Ac  is $< 7.5\times 10^{-5}\%$. However, the lower spallation cross section of $^{225}$Ra  at 438 MeV, along with smaller decay and chemical separation losses, limit the activity of the recovered $^{225}$Ac  to an order of magnitude lower than that obtained from radio-chemical separation of $^{225}$Ac  from an irradiated target^30^.

**Figure 2:**
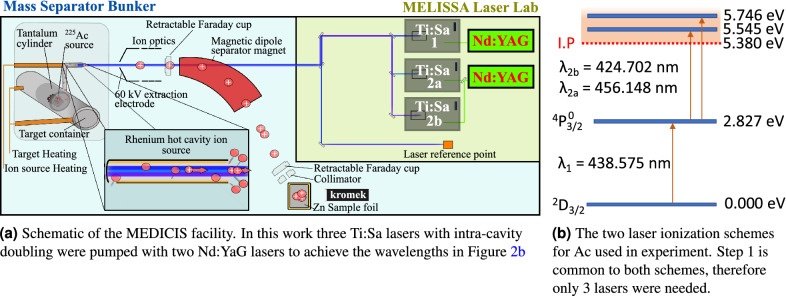
The experimental setup and ionization scheme used for $^{225}$Ac  collections at MEDICIS in this work.

In this work, we report on an alternative method that could be used to isolate $^{225}$Ac  from a high energy proton-irradiated U- or Th-based target, based on resonance ionization mass spectrometry (RIMS). This method is commonly used for the production of pure radioactive ion beams using a laser ion source followed by a mass separator^33^. In its working principle, RIMS can be applied to separate $^{225}$Ac  from an irradiated target as follows: The target is first irradiated, then loaded into an off-line mass separator as shown in Fig. 2a. the target container is heated to release the nuclear reaction products from the target as an atomic vapor. The vapor effuses to a hot cavity ion source that is illuminated by two or more spatially and temporally overlapped laser pulses. The frequencies of the lasers are tuned to resonantly induce electronic transitions in atoms of the element of interest to a level above the ionization potential. Ions are thus formed either from interaction with the lasers, or through loss of an electron to the interior surface of the hot cavity. These ions are accelerated across an electrostatic potential difference between the ion source and an extraction electrode. The resulting ion beam is then shaped and focused by electro-optical components, passed through a magnetic-dipole mass separator and implanted on a collection foil. This process is performed until depletion of the isotope from the target container which occurs when the ion beam current drops significantly below a nominal level. The main advantage of this method is its ability to produce beams of high isotopic purity thanks to the high mass resolution of dipole magnets at beam energies of tens of keV, combined with the elemental selectivity of resonant laser ionization.

Previously, RIMS has been performed on-line for the separation of $^{225}$Ac  from irradiated UC$_{x}$ for pre-clinical studies^34^. When produced and separated on-line, the instantaneous yield rate in number of ions per second of $^{225}$Ac  was measured^35^, however no collection efficiency could be calculated. Here, for the first time, the laser ionization efficiency and collection efficiency are measured for off-line RIMS of $^{225}$Ac  at CERN MEDICIS. Furthermore, the temperature required for $^{225}$Ac  release from a rhenium surface and a ThO$_{2}$  felt were determined.

## Methods

When RIMS is applied to separate an isotope from an irradiated target, the total collection efficiency, $\varepsilon _T$, can be split into contributions from the following sub-processes: diffusion of the atoms to the surface of target grains ($\varepsilon _{diff}$), effusion through the porous volume of the target and from the target to the ion source ($\varepsilon _{eff}$), ionization ($\varepsilon _{ion}$), separation through the mass separator ($\varepsilon _{sep}$) and beam transport ($\varepsilon _{trans}$). This efficiency decomposition is given by Eq. (1).

1 $$\begin{aligned} \varepsilon _T = \varepsilon _{diff} \varepsilon _{eff} \varepsilon _{ion} \varepsilon _{sep} \varepsilon _{trans}, \end{aligned}$$

In this work, the respective contributions of $\varepsilon _{ion}\varepsilon _{sep}\varepsilon _{trans}$ and $\varepsilon _{eff}\varepsilon _{ion}\varepsilon _{sep}\varepsilon _{trans}$ contributions to $\varepsilon _T$ were deduced. This was done by performing RIMS on two $^{225}$Ac  sources, each prepared in a different chemical environment. One chemical environment was a rhenium foil, and the other was a ThO$_{2}$  felt. In the former case, the efficiency contribution $\varepsilon _{ion}\varepsilon _{sep}\varepsilon _{trans}$ was probed. This is because upon heating, the deposited $^{225}$Ac  could effuse directly to the ion source. In the latter case, the deposited $^{225}$Ac  must also desorb and effuse through the ThO$_{2}$  pores before effusion to the ion source, thus incurring an extra efficiency term, $\varepsilon _{eff}$. In this campaign, three sources of $^{225}$Ac  were collected over two separate campaigns, corresponding to when the $^{225}$Ac  was initially deposited on the rhenium and ThO$_{2}$  respectively.

### Experimental conditions

Two separate samples of pure (>99.98%) $^{225}$Ac  were eluted from the Joint Research Centre Karlsruhe (JRC) $^{229}$Th generator using ion exchange and separation chromatography^36,37^. The first sample was deposited by evaporation of Ac(NO$_{3}$)$_{3}$ at 80 $^{\circ }$C onto a rhenium foil that clad the interior of a thick open-ended tantalum cylinder. This shall henceforth be referred to as the $^{225}$Ac$^{*}$  source. The second sample was prepared by the same evaporation process, but onto onto a small (1 g) nugget of ThO$_{2}$. This nugget was then placed in an identical open-ended tantalum cylinder clad with a rhenium foil. This shall be referred to as the $^{225}$Ac$^{*}$(ThO$_{2}$)  source. Prior to deposition, the activity of each Ac(NO$_{3}$)$_{3}$ solution was measured by gamma and alpha spectroscopy to be 30.0(1) MBq and 15.0(1) MBq for $^{225}$Ac$^{*}$  and $^{225}$Ac$^{*}$(ThO$_{2}$)  respectively. The two sources were shipped to the MEDICIS facility, where they were stored before RIMS was performed.

Each of the $^{225}$Ac  sources was separately loaded into the MEDICIS target container, a closed tantalum cavity that is resistively heated to operating temperature by applying a large current (up to 1000 A, but typically $\sim$ 600 A). The high temperature is required to vaporize the $^{225}$Ac. The tantalum target cavity is coupled to a rhenium ion source by means of a transfer line. The ion source is resistively heated by a current independent from that of the target (up to 400 A but typically $\sim$ 230 A) such that a plasma is created on the interior surface that generates a transversally-confining electric potential, while promoting guidance to the extraction field of the extraction electrode^38^. The ionization of $^{225}$Ac  was achieved through two different two-step ionization schemes that were first developed for laser spectroscopy^39,40^. They were employed simultaneously to drive transitions to auto-ionizing states shown in Fig. 2b. Since each of the schemes shared the same first step, only three lasers were required. The light in each case was generated with titanium-doped sapphire lasers (Ti:Sa) with intra-cavity second harmonic generation^41^. These were pumped with 532 nm light at a 10 kHz repetition rate from a commercial Nd:YAG laser (InnoLas NANIO 532-18-Y). A Ti:Sa cavity with a tunable grating provided the first step 438.575 nm light, while a Lyot filter and an etalon were used in the Ti:Sa cavities for selecting the 424.702 and 456.148 nm wavelengths for the second step transitions^42^. The bandwidth of the laser light was several Gigahertz. Following ionization, the ions were accelerated across a potential difference of 60 kV between the ion source and the extraction electrode. The resulting ion beam was then shaped and focused by a doublet of X-Y electrostatic deflectors and an einzel lens respectively. The beam then passed through a dipole magnet mass separator of resolving power MRP = 500 at mass number A = 100^43^. The mass-separated beam was finally implanted in a zinc film with a thickness of 500(5) nm that was vapor-deposited on a gold substrate 15 mm wide and 25 mm high. A different sample foil was used for each collection. During each collection, the sample foil was connected by means of electrical contact to a picoammeter from which data of the implanted beam current was recorded. Following implantation, the sample foils were recovered from the collection chamber and prepared for shipping to KU Leuven (Belgium) for analysis.

### Target temperature calibration

During the collections, there is no direct readout of temperature of the target container and ion source. Only the heating current supplied and the potential difference across each component are recorded. Therefore, before the collections began, the target container and ion source temperatures were calibrated using an optical pyrometer as a function of applied current up to nominal values of 625 A and 270 A respectively. However, during each collection of $^{225}$Ac, the target container was heated with currents that sometimes exceeded the maximum calibrated value, so an extrapolation was required. The temperature does not vary linearly with applied current in this high current regime, thus two extrapolation models were used, accounting for radiative and resistive power dissipation. The first model relates the calibrated temperature to supplied power, while the second relates it to supplied current. The two fitting functions are $P = aT + bT^4$ and $I = \frac{\alpha T}{T + \tau } + \frac{\beta T^4}{T + \tau }$ for the power and current models respectively. The reliability of these extrapolation functions was first tested on previous data sets for other target units with tantalum target containers coupled to rhenium ion sources. In these data sets, the current, power and temperature beyond nominal calibration values were measured. The reliability was tested by fitting only the data up to 600 A heating current, then measuring the residuals between model fit and data at higher currents. At currents up to 800 A, temperature differences of 30  $^{\circ }$C between the model fits and measured data were typical, with larger deviations (up to 80  $^{\circ }$C) observed for currents above 700 A in one of the data sets fitted by the power model. The temperatures predicted by each model typically differed by 100  $^{\circ }$C above currents of 700 A. The reliability of each model was similar, therefore the temperatures quoted in this work are the mean values of those predicted by the two models, with 1$\sigma$ uncertainties given by half the difference in temperature between each model.

### Collection conditions

#### $^{225}$Ac$^{*}$

**Figure 3:**
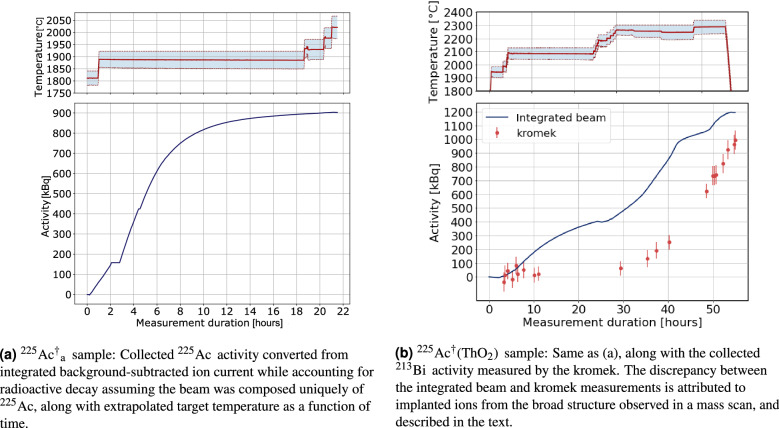
The target temperature and collected activity estimated during collection.

In November 2020, the $^{225}$Ac$^{*}$  source with an activity of 10.11(4) MBq was loaded into the MEDICIS target container. First, the transfer line was heated to 2170 $^{\circ }$C with a current of 290 A. A current was then applied to heat the target container. This was increased step-wise until 569 A, corresponding to a target container temperature of 1797 (42) $^{\circ }$C. The three lasers illuminated the ion source cavity at powers of 85 mW, 1.0 W and 0.9 W for steps 1, 2a and 2b respectively. Each atomic transition was found to be saturated at these laser powers. At this target temperature, a mass scan was performed. A beam current of 0.2 pA was measured on a Faraday cup at the magnetic field strength calibrated to mass A = 225. These conditions were maintained for 1.75 h. Subsequently, the target current was increased to 602 A (1890 (40) $^{\circ }$C), where the collection proceeded for a duration of 17.58 h. The current was finally increased to 622 A (1930(50) $^{\circ }$C) and 642 A (1975(45) $^{\circ }$C) for a duration of 1.9 h and 0.75 h respectively. At certain intervals during the collection, tests to measure the laser ionization enhancement were performed which lead to momentary dips in the ion current, as seen in Fig. 6a. Furthermore, the Faraday cup downstream of the mass separator was occasionally actuated. During these instances the background ion current on the picoammeter connected to the sample was measured. It was found that the background current decreased over time such that negative values of ion current were observed, even while $^{225}$Ac  was being implanted (Fig. 6a). This background was attributed to alpha emission from the implanted $^{225}$Ac  and its daughter nuclei leading to an effective negative ion current resulting from the nuclear recoil. During the collection, the activity of the implanted $^{225}$Ac  was monitored by measuring the count rate of the 440 keV $^{213}$Bi gamma decay using a portable cadmium zinc telluride (CZT) detector, GR1, from Kromek$^{\copyright }$. This detector has been thoroughly characterized for use in the MEDICIS collection chamber^44^. The collection under these conditions was stopped when the count rate was observed to no longer increase. The end of collection activity of this $^{225}$Ac  sample was estimated by the Kromek GR1 and integrated ion current to be approximately 1 MBq. A graphical representation of target temperature as well as post-analysed integrated beam current in units of equivalent $^{225}$Ac  activity is shown in Fig. 3a. This collected sample shall be referred to as $^{225}$Ac$^{\dagger }_{\textrm{a}}$.

A new clean foil was then placed in front of the beam. The collection proceeded with higher target currents of 660 A, 680 A and 720 A applied sequentially for 1.92, 19.42 and 4.67 h respectively. This was done to investigate whether more Ac could be extracted at higher target temperatures. It was estimated that this sample had an activity of 180 kBq of $^{225}$Ac at the end of collection from Kromek GR1 measurements. This sample shall henceforth be referred to as $^{225}$Ac$^{\dagger }_{\textrm{b}}$.

#### $^{225}$Ac$^{*}$(ThO$_{2}$)

In December 2020, the $^{225}$Ac$^{*}$(ThO$_{2}$)  source was placed in the MEDICIS target container in the same configuration as the $^{225}$Ac$^{*}$  source. The $^{225}$Ac$^{*}$(ThO$_{2}$)  activity was 9.30(7) MBq at the start of the collection. The lasers were also calibrated and aligned to the ion source to replicate the previous conditions. Laser output powers of 85 mW, 0.9 W, and 1.1 W were attained for steps 1, 2a and 2b respectively. The target current was incrementally increased to 700 A (2090(39) $^{\circ }$C). However, Kromek measurements of $^{213}$Bi activity indicated that little $^{225}$Ac  was being implanted, as shown in Fig. 3b. After 24 h, a mass scan was performed, which showed a broad structure extending over masses 179 until mass 244. The peak of this structure was at mass 195. This structure lead to an ion beam of approximately 5 pA at mass 225 at this time during the collection. These ions were the cause of the increasing integrated ion current shown in Fig. 3b while no significant increase in the $^{213}$Bi  activity was observed. After the mass scan, the current was then increased incrementally to 790 A (2264(39) $^{\circ }$C), whereupon it was left for 18.4 h, during which $^{225}$Ac  was implanted. For the final 5.8 h, the current was further increased to 810 A (2288(50) $^{\circ }$C), during which $^{225}$Ac  continued to be implanted. The collection was stopped after 52 h due to facility constraints, while the observed collected $^{225}$Ac  activity was still increasing, contrary to the previous collection from the $^{225}$Ac$^{*}$source. This $^{225}$Ac  sample shall be referred to as $^{225}$Ac$^{\dagger }$(ThO$_{2}$). Once the collection ended, another mass scan was immediately performed. At this temperature, a broad peak was observed centred on mass 217 which is consistent with Th$^{+}$ from fragmentation of ThO$^{+}$ in the field-free region between the extraction electrode and mass separator. The broad structure was still present, but with much lower beam intensity at the masses observed.

### Characterisation of collected samples

At KU Leuven, the activities of the three samples $^{225}$Ac$^{\dagger }_{\textrm{a}}$, $^{225}$Ac$^{\dagger }_{\textrm{b}}$  and $^{225}$Ac$^{\dagger }$(ThO$_{2}$)  were measured using alpha, gamma and gamma-gamma coincidence spectroscopy in order to determine the end of collection (EOC) activity. The alpha spectroscopy was performed in high vacuum ($< 10^{-5}$ mbar) with a passivated implanted planar silicon (PIPS) detector. Dead-time, resolution broadening, and geometric efficiency for detecting alpha radiation from each daughter in the $^{225}$Ac  decay chain due to nuclear recoil were accounted for. For the gamma spectroscopy measurements, the samples were contained in a small glass vial to contain the recoiling daughter nuclei. The vials were each placed 25 cm above a co-axial high purity germanium (HPGe) detector on a hollow plastic mount. The sample and detector were shielded from background radiation with a lead castle. Spectra were recorded over measurement periods of approximately 6 h. From these data, the activities of $^{213}$Bi  and $^{221}$Fr  at these times were calculated from their prominent $\gamma$-emissions at 218 keV and 440 keV respectively. The gamma-gamma spectroscopy was performed using two co-axial HPGe detectors at 90$^{\circ }$from each other. The coincidence count rate gated on 465 ± 5 keV and 1567 ± 5 keV energy peaks following the $^{209}$Tl  $\beta ^{-}$ decay within a 200 ns coincidence window was measured. The count rates within the aforementioned energy windows in each detector were also recorded. The $^{209}$Tl  activity was then calculated from the quotient of the product of the single count rates to the coincidence count rates up to branching intensity factors. This method is favourable as the calculated activity depends neither on the detector efficiency nor the geometric efficiency, which changes for each $\alpha$-decay recoil daughter in the $^{225}$Ac  decay chain. Further details on all of the decay spectroscopy measurements are found in the supplementary material^45^.

After the decay spectroscopy campaign, the two most active samples, $^{225}$Ac$^{\dagger }_{\textrm{a}}$  and $^{225}$Ac$^{\dagger }$(ThO$_{2}$), were shipped to the Belgian Nuclear Research Centre SCK CEN. There, the $^{225}$Ac-implanted Zn layer was dissolved in 10 mL of 1 M HNO$_{3}$ acid at 30 $^{\circ }$C for 30 min. In these tests, >90% of $^{225}$Ac  was recovered in solution, and was therefore implanted in the 500 nm thick zinc layer. This fraction of $^{225}$Ac  is thus able to be used for further radio-labelling. Alpha-decay spectroscopy was later performed on the gold sample foils on which the dissolution tests had been performed. It was found that some $^{225}$Ac  was present on the gold foil. This could be due to ion channeling of the $^{225}$Ac  through the zinc film.

## Results

### Collection efficiencies

**Figure 4:**
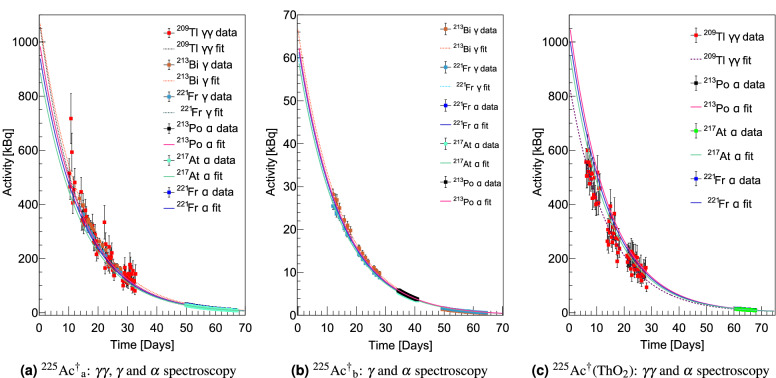
The activities of the three $^{225}$Ac  sources measured by decay spectroscopy.

In order to determine the collection efficiency of $^{225}$Ac, the following equation was used,

2 $$\begin{aligned} \varepsilon _{T} =\frac{A_f(t_f)}{A_t(t_i)}, \end{aligned}$$

where $A_t(t_i)$ is the $^{225}$Ac  activity in the source loaded into the target at the time the collection begins and $A_f(t_f)$ is the $^{225}$Ac  activity on the collection foil when the collection ends. In this work, $A_t(t_i)$ was calculated using the radioactive decay law with the reference activity and time given by the alpha and gamma spectroscopy performed at the JRC. The EOC activity, $A_f(t_f)$, was determined using back extrapolation of the activities measured by decay spectroscopy at KU Leuven. The EOC activities for $^{225}$Ac$^{\dagger }_{\textrm{a}}$, $^{225}$Ac$^{\dagger }_{\textrm{b}}$  and $^{225}$Ac$^{\dagger }$(ThO$_{2}$)  were 962(22), 62.3(23) and 925(77) kBq respectively. The extrapolated decay curves are shown in Fig. 4. These activities are systematically lower than those recorded during the collections by the Kromek GR1 spectrometer, whose purpose was only indicative. This is due to the detection range of the Kromek GR1 detector which is positioned within the collection chamber. It is thus sensitive to decays of $^{225}$Ac  and daughters that are implanted elsewhere in the chamber, such as the collimator or neighboring foils. The collection efficiency of $^{225}$Ac$^{*}$, given by $\varepsilon _{ion}\varepsilon _{sep}\varepsilon _{trans} =$ $\varepsilon _T$($^{225}$Ac$^{\dagger }_{\textrm{a}}$) $+ \varepsilon _T$($^{225}$Ac$^{\dagger }_{\textrm{b}}$) was determined to be 10.1(2)%.

In a similar manner, the collection efficiency was calculated for $^{225}$Ac$^{*}$(ThO$_{2}$)  to be $\varepsilon _T$($^{225}$Ac$^{\dagger }$(ThO$_{2}$)) = 9.9(8)%. This corresponds, however, to the case in which the cumulative implanted activity was still rising. Thus the collection efficiency would be higher had the collection continued until the $^{225}$Ac$^{\dagger }$(ThO$_{2}$)  source was depleted, as was the case in the collection of $^{225}$Ac$^{*}$.

### Ionization efficiency

The ionization efficiency was determined from the collection efficiency of $^{225}$Ac$^{*}$using Eq. (1) with neither diffusion nor effusion contributions. The beam transport efficiency, $\varepsilon _{trans}$ was calculated by taking the ratio of the current of the separated beam on the collection foil to that on a retractable Faraday cup positioned immediately after the mass separator. These measurements were taken at 4.28 and 6 h after the start of collection, when the separated ion beam current at mass 225 was sequentially measured on the Faraday cup and then on the collection foil. The transport efficiency was thus determined to be $\varepsilon _{trans} = 87(3)$  %. Further losses due to practical separator operation were also taken into consideration, with a corresponding efficiency term, $\varepsilon _{op}$. During the collection of $^{225}$Ac$^{*}$, laser optimization was being performed while the target was at 1810 $^{\circ }$C, for a duration of 2 h before the start of the collection. Furthermore, a mass scan was performed after heating the target to 1890 $^{\circ }$C. In these instances the beam was not implanted on the collection foil. The beam current over these times was estimated based on Monte-Carlo interpolation of recorded ion current at the same temperature, shown in Fig. 6a. The negative background current due to the accumulation of $\alpha$-emitting nuclides on the collection foil was subtracted. The resulting background subtracted ion current was integrated and converted to equivalent EOC activities while accounting for decay loss. Activity losses of $A_{loss}^{(0)}(t_f) = 182(13)$ kBq and $A_{loss}^{(1)}(t_f) = 115(3)$ kBq were obtained for the mass scan and laser optimisation periods respectively.

$\varepsilon _{op}$ was determined from Eq. (3) by taking the ratio of the total collected activity to the sum of the total collected activity and that lost due to the reasons given above.

3 $$\begin{aligned} \varepsilon _{op} = \frac{A(^{225}Ac^{\dagger }_{\textrm{a}})(t_f^a) + A(^{225}Ac^{\dagger }_{\textrm{b}})(t_f^b)}{A(^{225}Ac^{\dagger }_{\textrm{a}})(t_f^a) + A(^{225}Ac^{\dagger }_{\textrm{b}})(t_f^b)+ A_{loss}^{(0)}(t_f^a) + A_{loss}^{(1)}(t_f^a) } = 77.5(9)\% \end{aligned}$$

Assuming that the losses due to mass separator acceptance of the beam from the ion source were negligible ($\varepsilon _{sep} = 100\%$), the ionization efficiency which comprises both laser and surface ionization mechanisms was then deduced as $\varepsilon _{ion} = \frac{\varepsilon _{T}}{\varepsilon _{trans}\varepsilon _{op} }= 15.1(6)\%$.

## Discussion

### Ion source performance

The ionization efficiency of 15.1(6)% calculated for the $^{225}$Ac$^{*}$  source is compared in Table 1 to that of RIMS of other actinides and lanthanides in similar experimental conditions. In these previous works the ionization efficiencies were calculated using the same experimental technique as in this work. It is notable that the post-separation ion current for the collection of $^{225}$Ac  in this work is approximately three orders of magnitude lower than that of the actinides in Table 1 while still having a lower ionization efficiency. Furthermore, post-separator ion currents above the nanoampere range were observed at MEDICIS for highly efficient resonant ionization of $^{167}$Tm using an identical ion source. This suggests that the ionization efficiency in this work is not limited by high-throughput effects that lead to ion-electron recombination on the hot cavity surface or repression of the potential from the extraction electrode with increasing ion current density^38,46,47^.

**Table 1:** Ionization efficiencies and maximum post-magnetic-separation ion currents for some previously studied actinides and lanthanides.

Facility	Isotope	Ion source dimensions $\varnothing$ $\times$ L (mm)	Typical ion current (nA)	Ionization efficiency (%)
ORNL^48^	$^{232}$Th	$3 \times 30$	50	38.6
ORNL^49^	$^{242}$Pu	$3 \times 30$	10	51.1
RISIKO (Mainz)^50^	$^{177}$Lu	$2.5 \times 35$	40	52
RISIKO (Mainz)^51^	$^{159}$Tb	$2.5 \times 35$	6	53
RISIKO (Mainz)^52^	$^{165}$Ho	$2.2 \times 30$	50	32
RISIKO (Mainz)^53^	$^{164}$Dy	–	1	25
IRIS^54^	$^{nat}$Yb	$1.5 \times 50$	–	35
MEDICIS^55^	$^{153}$Sm	$3 \times 34$	–	12.7
MEDICIS^56^	$^{167}$Tm	$3 \times 34$	50	55
MEDICIS^57^	$^{155}$Tb	$3 \times 34$	10–15	1–6
MEDICIS	$^{225}$Ac	$3 \times 34$	0.01	15.1

In this work and that performed at ORNL and RISIKO in Table 1, the species of interest were prepared in samples of high isotopic purity, rather than being extracted from irradiated targets, so the ion current density in the ion source from other isotopes was low. This further indicates that the lower ionization efficiency of $^{225}$Ac  at MEDICIS may be attributable to the oscillator strength of the stimulated electronic transitions used for photo-ionization, shown in Fig. 2, rather than ion source limitations. This is in contrast to the case of $^{155}$Tb extraction at MEDICIS, where the ionization efficiency is typically only 1-6% for extraction from an irradiated target due to ionization of the more volatile Gd and Dy isotopes released from the target that saturate the ion source. For comparison, this is an order of magnitude lower than that reported at RISIKO for a pure $^{159}$Tb source where the ion source efficiency was not limited by high throughput effects. An increase of the $^{225}$Ac  ionization efficiency could then be sought by performing a survey of second step transitions to Rydberg or auto-ionizing states with greater intensity. However, this would require further confirmation as the ion source performance also depends on other factors that affect the plasma sheath potential. For example, atoms of the target, target unit and ion source themselves may be vaporized and ionized. Such effects would become more important at the high target and ion source temperatures needed for $^{225}$Ac  extraction, which is more than is typically used for other isotopes, including those in Table 1.

Moreover, in future collections of $^{225}$Ac  from irradiated targets, the 15.1(6)% ionization efficiency may not be achieved for equivalent target and ion source temperature and laser parameters. This is due to the increased ion current in the ion source as atoms from the irradiated target effuse into the ion source cavity, as previously described for the Tb case. If the ionization efficiency of $^{225}$Ac  is found to be reduced by such high throughput effects, an alternative approach for $^{225}$Ac  separation from an irradiated target could be employed. First, radio-chemical separation could be performed, which is known to have high $^{225}$Ac  radiochemical yield^28–30^. the recovered $^{225}$Ac  fraction (containing $^{227}$Ac  and $^{225}$Ac ) could then be dried and evaporated on a rhenium foil. the RIMS method could then be performed on this sample. Such an approach would reduce the overall ion load. This is because far fewer potential contaminant species would be present in the target container and a lower target temperature would be required for $^{225}$Ac  release, thus the vaporization rate of contaminant species would also be reduced. It is also possible that a new design of hot cavity laser ion source optimized for high throughput beams could overcome such potential limitations. This would mean that RIMS could be performed on irradiated targets directly without suffering ionization efficiency reduction due to ion load issues. Further systematic studies of the ionization efficiency of Ac as a function of ion source temperature and total ion current are thus critical to investigate these effects.

### Optimal target temperature for $^{225}$Ac  extraction from ThO$_{2}$

In future experiments at MEDICIS, $^{225}$Ac  will be extracted from irradiated ThO$_{2}$  targets. It is therefore important to determine the target temperature at which $^{225}$Ac  is vaporized sufficiently to form an ion beam intense enough to collect a large fraction of the available $^{225}$Ac. Data obtained from the Kromek GR1 detector shown in Fig. 3b indicated that a minimum target temperature, $T_{tar}$ of 2264(39) $^{\circ }$C was required for significant $^{225}$Ac  extraction from the $^{225}$Ac$^{*}$(ThO$_{2}$)  source. This is much higher than the 1890(40) $^{\circ }$C required to extract $^{225}$Ac  from the $^{225}$Ac$^{*}$  source.

As a first investigation of this effect, thermo-mechanical simulations were performed to obtain steady-state temperature distributions of the target-ion source unit using ANSYS 2019 R3^58^. The simulations were performed for cases where the tantalum container was placed at different positions within the target container, and cases where ThO$_{2}$  nuggets of varying sizes were present inside it, including those corresponding to the $^{225}$Ac$^{*}$  and $^{225}$Ac$^{*}$(ThO$_{2}$)  sources. The results show that the relative temperature difference of the Tantalum cylinder changes by less than 3% for positions about the centre of the target container. Furthermore, the simulation results show that the ThO$_{2}$  temperature does not significantly differ from that of the tantalum cylinder. This suggests that the increase in target temperature required for $^{225}$Ac  release from ThO$_{2}$  was not due to a different temperature of the $^{225}$Ac  source itself when the ThO$_{2}$  was present in the container, but that the physical temperature required for $^{225}$Ac  release from ThO$_{2}$  was higher^59^.

**Figure 5:**
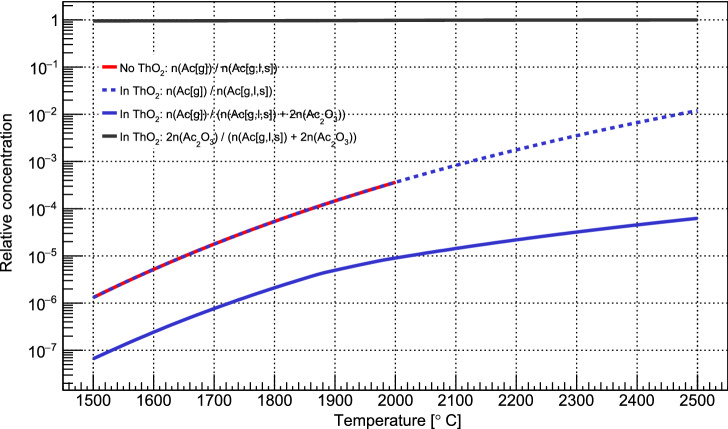
The relative equilibrium concentration of Ac species in a ThO$_{2}$  environment and in a pure environment. Most Ac is locked up in Ac$_{2}$O$_{3}$, while the equilibrium gas phase concentration Ac(g) increases exponentially with temperature, but at different rates. The simulations also account for the liquid and solid phases, labeled (l) and (s) respectively.

An explanation for the observed temperature required for $^{225}$Ac  release from ThO$_{2}$  was then sought by simulating the equilibrium concentrations of $^{225}$Ac  phases and species both with and without a large excess of ThO$_{2}$  at different temperatures. The program HSC chemistry was used^60^, which generates equilibrium concentrations of species by performing Gibbs free energy minimization. This calculation was only qualitative, however, as data on formation enthalpies of some Ac-containing compounds that could be formed are unknown and therefore not considered in the simulation. Still, the results show that when there is a large excess of ThO$_{2}$  at $T_{tar}>$ 1800 $^{\circ }$C, the refractory molecule Ac$_{2}$O$_{3}$ forms and accounts for more than 95% of Ac in the system. Furthermore, its relative equilibrium concentration grew with increasing $T_{tar}$. The relative gas-phase concentration of Ac also rapidly increased with $T_{tar}$, but at a slower rate than when no ThO$_{2}$  was present, as shown in Fig. 5. These results give a physical grounding to our observation of the high-temperature threshold ( $\approx 2260(40)$ $^{\circ }$C) for $^{225}$Ac  extraction from ThO$_{2}$. A higher temperature is required to increase the Ac(g) concentration to a level that leads to an appreciable extracted ion current.

### $^{225}$Ac  extraction rate

Finally, the temperature-dependent time scale of $^{225}$Ac  extraction comprising vaporization and desorption from the rhenium foil and effusion to the ion source was estimated during the collection of $^{225}$Ac$^{\dagger }_{\textrm{a}}$. Firstly, the background-free ion current was calculated while the target temperature was stable at $T_{tar} =$1890 $^{\circ }$C. The ion current on the sample foil recorded by the picoammeter while the Faraday cup was blocking the beam upstream was measured. The background was defined by interpolation of these beam current data using a sigmoid function. This was then subtracted from the recorded ion beam current while no Faraday cup was actuated upstream of the sample foil. Fitting of the background-subtracted ion current was performed with a series of exponential functions based on the general solution to the diffusion equation, describing the transport of radioisotopes from solid materials^61^. Here, it is implicitly assumed that the $^{225}$Ac  beam current is proportional to the release rate of atomic $^{225}$Ac  up to a constant ionization efficiency term. It was observed that the time behaviour of the beam current in this case was sufficiently modelled by a single exponential function with a constant background. Attempts to fit with further exponential terms resulted in equal rate parameters in the argument of each exponential term, rendering them redundant.

**Figure 6:**
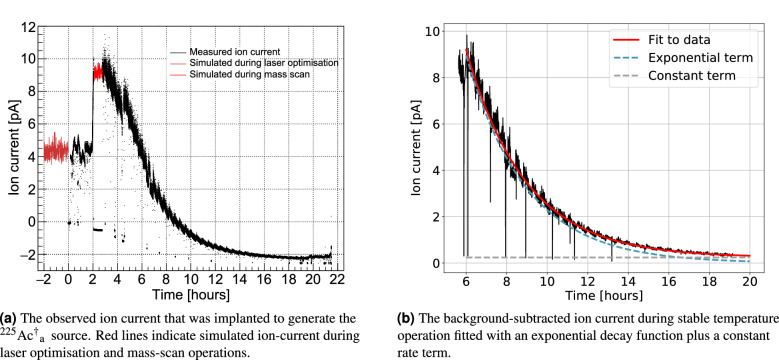
The ion current during collection of the $^{225}$Ac$^{\dagger }_{\textrm{a}}$  sample.

A general equation for the ion flux along the longitudinal z axis of the ion source can be given by Eq. (4), which is proportional to a general solution to the diffusion equation. Explicit dependencies on target and ion source temperatures, $T_{tar}$ and $T_{ion}$ are indicated.

4 $$\begin{aligned} I(z, t, T_{ion}, T_{tar}) = \varepsilon _{ion}(z, T_{ion}) \frac{dn(z,t,T_{tar})}{dt} = \sum _{i = 1}^{\infty } \, \phi _i(z,T_{ion}, T_{tar}) \, e^{-\lambda _i(T_{tar})t}. \end{aligned}$$

The fit performed in Fig. 6b only takes the first exponential term into account. The parameter $\lambda _1$ that broadly represents the release rate of $^{225}$Ac  in the specific conditions of the target and ion source at this time was deduced. A value of $\lambda _1 = 0.34$ h$^{-1}$ corresponding to a time constant of $t_{1/2} = 2.03$ h was obtained. The fit in Fig. 6b also includes a constant term. This may account for a component of the $^{225}$Ac  ion current formed from Ac that is vaporized and thus released at a much slower rate, for example, from a target cold-spot. While this effective release time is only valid for target temperature $T_{tar} =$1890 $^{\circ }$C, the release would be faster with increasing temperature, due to faster effusion^62^, as well as increase in $^{225}$Ac  vapor pressure. Thus, at higher target temperatures, $^{225}$Ac  collections could be performed until source depletion faster than in this experiment. On the other hand, the higher ion loads under such conditions could also lead to reduction in ion source efficiency. The target temperature should therefore be selected to sufficiently release $^{225}$Ac  while not overloading the ion source. It was also observed in in collection of $^{225}$Ac$^{\dagger }$(ThO$_{2}$)  that the presence of ThO$_{2}$  significantly reduced $^{225}$Ac  release at a given temperature. A negligible amount of $^{225}$Ac  was collected at the temperature $T_{tar} =$2090 $^{\circ }$C, which is more than what was necessary for collection of $^{225}$Ac$^{\dagger }_{\textrm{a}}$. This was shown in the previous discussion to be due to reduced $^{225}$Ac  vapor pressure for a given target temperature. Therefore, the presence of other species that affect the chemical environment in the target unit also have a large impact on the rate of $^{225}$Ac  release.

## Conclusion

A collection efficiency of 10.1(2)% for laser ionized and mass separated $^{225}$Ac  with an associated ionization efficiency of 15.1(6)% has been achieved at MEDICIS using two simultaneous two-step resonant laser ionization schemes. It has been demonstrated that a similar collection efficiency is achievable even when the $^{225}$Ac  is evaporated onto a ThO$_{2}$  target matrix, but at a higher target temperature of 2260(40) $^{\circ }$C compared to 1890(40) $^{\circ }$C. The collection efficiency of pure $^{225}$Ac  is of a similar order to the ratio of pure $^{225}$Ac  produced by second pass chemical separation, to that directly produced in target at TRIUMF^30^. This result suggests that laser ionization and mass separation is a promising method for producing pure $^{225}$Ac, that could complement radio-chemical methods, depending on the infrastructure of potential $^{225}$Ac  production facilities. Investigations into $^{225}$Ac  collection efficiency from an irradiated ThO$_{2}$  target to establish the overall collection efficiency are ongoing. The collection efficiency is expected to decrease due to intra-grain diffusion losses as well as a lower ionization efficiency due to increased ion load from the many contaminants co-produced in-target. Furthermore, a study of the $^{227}$Ac  contamination in samples following RIMS of $^{225}$Ac  from an irradiated ThO$_{2}$  target is ongoing to experimentally determine the isotopic purity of $^{225}$Ac  collected by this method. These efficiency and purity determinations will provide further insight into the suitability of RIMS for $^{225}$Ac  extraction for medical use.

## Supplementary Information

Supplementary Information.

## Data Availability

The datasets analysed during the current study are available at https://zenodo.org/record/7296558.

## References

[1] Leddy ET, Weatherwax JL (1925). The Roentgen treatment of advanced cancer. Radiology.

[2] Barnett GC (2009). Normal tissue reactions to radiotherapy: Towards tailoring treatment dose by genotype. Nat. Rev. Cancer.

[3] Hanahan D, Weinberg RA (2011). Hallmarks of cancer: The next generation. Cell.

[4] Lambert AW, Pattabiraman DR, Weinberg RA (2017). Emerging biological principles of metastasis. Cell.

[5] Geiger TR, Peeper DS (2009). Metastasis mechanisms. Biochim. Biophys. Acta (BBA) Rev. Cancer.

[6] Gudkov SV, Shilyagina NY, Vodeneev VA, Zvyagin AV (2016). Targeted radionuclide therapy of human tumors. Int. J. Mol. Sci..

[7] Jadvar H (2017). Targeted radionuclide therapy: An evolution toward precision cancer treatment. Am. J. Roentgenol..

[8] Pouget J-P, Lozza C, Deshayes E, Boudousq V, Navarro-Teulon I (2015). Introduction to radiobiology of targeted radionuclide therapy. Front. Med..

[9] Nikjoo H (2016). Radiation track, DNA damage and response—A review. Rep. Prog. Phys..

[10] Desouky O, Ding N, Zhou G (2015). Targeted and non-targeted effects of ionizing radiation. J. Radiat. Res. Appl. Sci..

[11] Chen H (2019). Integrin $$\alpha$$v$$\beta$$3-targeted radionuclide therapy combined with immune checkpoint blockade immunotherapy synergistically enhances anti-tumor efficacy. Theranostics.

[12] Choi J (2018). Combined VLA-4-targeted radionuclide therapy and immunotherapy in a mouse model of melanoma. J. Nucl. Med..

[13] Demaria S, Golden EB, Formenti SC (2015). Role of local radiation therapy in cancer immunotherapy. JAMA Oncol..

[14] Radchenko V (2021). Production and supply of $$\alpha$$-particle-emitting radionuclides for targeted $$\alpha$$-therapy. J. Nucl. Med..

[15] Pommé S (2012). Measurement of the 225Ac half-life. Appl. Radiat. Isot..

[16] Kossert K, Takács MP, Nähle O (2020). Determination of the activity of 225Ac and of the half-lives of 213Po and 225Ac. Appl. Radiat. Isot..

[17] Morgenstern A, Apostolidis C, Bruchertseifer F (2020). Supply and clinical application of Actinium-225 and Bismuth-213. Seminars in Nuclear Medicine.

[18] Perron R, Gendron D, Causey PW (2020). Construction of a thorium/actinium generator at the Canadian Nuclear Laboratories. Appl. Radiat. Isot..

[19] Boden S (2017). Thorium-229 quantified in historical Thorium-228 capsules. Appl. Radiat. Isot..

[20] Yan W (2020). Mining medical isotopes from nuclear waste. ACS Central Sci..

[21] Bruchertseifer F, Kellerbauer A, Malmbeck R, Morgenstern A (2019). Targeted alpha therapy with Bismuth-213 and Actinium-225: Meeting future demand. J. Label. Compd. Radiopharm..

[22] Robertson AK, Ramogida CF, Schaffer P, Radchenko V (2018). Development of 225Ac radiopharmaceuticals: TRIUMF perspectives and experiences. Curr. Radiopharm..

[23] Ermolaev S (2012). Production of Actinium, Thorium and Radium isotopes from natural thorium irradiated with protons up to 141 MeV. Radiochim. Acta.

[24] Weidner J (2012). 225Ac and 223Ra production via 800 MeV proton irradiation of natural thorium targets. Appl. Radiat. Isot..

[25] Griswold JR (2016). Large scale accelerator production of 225Ac: Effective cross sections for 78–192 MeV protons incident on 232Th targets. Appl. Radiat. Isot..

[26] Harvey J (2010). Production of Actinium-225 via high energy proton induced spallation of Thorium-232. Applications of High Intensity Proton Accelerators.

[27] John K (2019). US DOE tri-lab research and production effort to provide accelerator-produced 225Ac for radiotherapy: 2019 update. J. Med. Imaging Radiat. Sci..

[28] Aliev R (2014). Isolation of medicine-applicable actinium-225 from thorium targets irradiated by medium-energy protons. Solvent Extract. Ion Exch..

[29] Radchenko V (2015). Application of ion exchange and extraction chromatography to the separation of actinium from proton-irradiated thorium metal for analytical purposes. J. Chromatogr. A.

[30] Robertson AK (2020). 232Th-spallation-produced 225Ac with reduced 227Ac content. Inorg. Chem..

[31] Euratom. Recommendations of the Group of Experts established under the terms of Article 31 of the Euratom Treaty. Tech. Rep. (European commission, 2000).

[32] Junior, J. A. O. *et al.* Report on joint IAEA-JRC Workshop Supply of Actinium-225. Tech. Rep. (IAEA, 2018).

[33] Fedosseev V (2017). Ion beam production and study of radioactive isotopes with the laser ion source at isolde. J. Phys. G Nucl. Part. Phys..

[34] Ramogida CF (2019). Evaluation of polydentate picolinic acid chelating ligands and an $$\alpha$$-melanocyte-stimulating hormone derivative for targeted alpha therapy using ISOL-produced 225 Ac. EJNMMI Radiopharm. Chem..

[35] Kunz P (2018). Medical isotopes from Isac actinide targets. Prog. Nucl. Sci. Technol..

[36] Apostolidis C, Molinet R, Rasmussen G, Morgenstern A (2005). Production of Ac-225 from Th-229 for targeted $$\alpha$$ therapy. Anal. Chem..

[37] Zielinska B, Apostolidis C, Bruchertseifer F, Morgenstern A (2007). An improved method for the production of Ac-225/Bi-213 from Th-229 for targeted alpha therapy. Solvent Extract. Ion Exch..

[38] Kirchner R (1990). On the thermoionization in hot cavities. Nucl. Instrum. Methods Phys. Res. Sect. A Accel. Spectrom. Detect. Assoc. Equip..

[39] Raeder S (2013). In-source laser spectroscopy developments at Trilis—Towards spectroscopy on actinium and scandium. Hyperfine Interact..

[40] Ferrer R (2017). Towards high-resolution laser ionization spectroscopy of the heaviest elements in supersonic gas jet expansion. Nat. Commun..

[41] Gadelshin V (2020). MELISSA: Laser ion source setup at CERN-MEDICIS facility. Blueprint. Nucl. Instrum. Methods Phys. Res. Sect. B Beam Interact. Mater. Atoms.

[42] Rothe S, Marsh B, Mattolat C, Fedosseev V, Wendt K (2011). A complementary laser system for ISOLDE RILIS. J. Phys. Conf. Ser..

[43] Palenzuela, Y. M. *et al.* The CERN-MEDICIS isotope separator beamline. *Front. Med.* **8** (2021). 10.3389/fmed.2021.689281 PMC8450321 34552941

[44] Christodoulou, P. An in situ gamma-spectrometry system for the characterization of non-conventional radionuclides for medical research. https://cds.cern.ch/record/2732064 (2020).

[45] Johnson, J. & Michael, H. Supplementary material to Resonant Laser Ionization and Mass Separation of 225Ac. *Nat. Sci. Rep.* (2022). 10.1038/s41598-023-28299-4 PMC9873802 36693865

[46] Fedosseev V (2017). Ion beam production and study of radioactive isotopes with the laser ion source at ISOLDE. J. Phys. G Nucl. Part. Phys..

[47] Latuszynski A, Raiko V (1975). Studies of the ion source with surface-volume ionization. Nucl. Instrum. Methods.

[48] Liu Y, Stracener D (2020). High efficiency resonance ionization of thorium. Nucl. Instrum. Methods Phys. Res. Sect. B Beam Interact. Mater. Atoms.

[49] Galindo-Uribarri A, Liu Y, Romero Romero E, Stracener DW (2021). High efficiency laser resonance ionization of plutonium. Sci. Rep..

[50] Gadelshin VM (2019). Measurement of the laser resonance ionization efficiency for lutetium. Radiochim. Acta.

[51] Gadelshin VM (2021). Terbium medical radioisotope production: Laser resonance ionization scheme development. Front. Med..

[52] Schneider F (2016). Resonance ionization of holmium for ion implantation in microcalorimeters. Nucl. Instrum. Methods Phys. Res. Sect. B Beam Interact. Mater. Atoms.

[53] Studer D, Dyrauf P, Naubereit P, Heinke R, Wendt K (2017). Resonance ionization spectroscopy in dysprosium. Hyperfine Interact..

[54] Alkhazov G (1991). Application of a high efficiency selective laser ion source at the iris facility. Nucl. Instrum. Methods Phys. Res. Sect. A Accel. Spectrom. Detect. Assoc. Equip..

[55] Van de Voorde M (2021). Production of Sm-153 with very high specific activity for targeted radionuclide therapy. Front. Med..

[56] Talip, Z. *et al.* Efficient production of high specific activity 167Tm at PSI and CERN-MEDICIS. *Laboratory of Radiochemistry* 52 (2020).

[57] Duchemin, C. *et al.* CERN-MEDICIS: A review since commissioning in 2017. *Front. Med.* **8** (2021). 10.3389/fmed.2021.693682 PMC8319400 34336898

[58] Ansys Inc. ANSYS R3. https://www.ansys.com/ (2019).

[59] Hurier, S. MED024—ANSYS Simulation for Ac-225 collections. *CERN document server*. https://cds.cern.ch/record/2767597 (2021).

[60] Metso Outotec. HSC Chemistry 10.0.04. https://www.hsc-chemistry.com/ (2020).

[61] Fujioka M, Arai Y (1981). Diffusion of radioisotopes from solids in the form of foils, fibers and particles. Nucl. Instrum. Methods Phys. Res..

[62] Mehrer H (2007). Diffusion in Solids: Fundamentals, Methods, Materials, Diffusion-Controlled Processes.

