# Supplementary material for: Resonant laser ionization and mass separation of 225Ac
Source: Sci Rep. 2023 Jan 24;13:1347. doi: 10.1038/s41598-023-28299-4 (PMC9873802; doi:10.1038/s41598-023-28299-4)
Supplement: Supplementary file 1 — Supplementary Information. [file 41598_2023_28299_MOESM1_ESM.zip › compiled_PDF_version/Actinium_supplement-2.pdf]

Resonant Laser Ionization and Mass Separation of ^{225}Ac: Supplementary material

Jake D. Johnson[1 ✉], **Michael Heines**[1], **Frank Bruchertseiffer**[2], **Eric Chevallay**[3], **Thomas E. Cocolios**[1], **Charlotte Duchemin**[1,3], **Kristof Dockx**[1], **Stephan Heinitz**[4], **Reinhard Heinke**[1,3], **Sophie Hurier**[1,4], **Laura Lambert**[3], **Benji Leenders**[4,5], **Hanna Skliarova**[4], **Thierry Stora**[3], **and Wiktoria Wojtaczka**[1]

[1] KU Leuven, IKS, Leuven, 3000, Belgium
[2] JRC, Karlsruhe, Germany
[3] CERN, Geneva, 1951, Switzerland
[4] Belgian Nuclear Research Centre SCK CEN, Mol, Belgium
[5] Universiteit Gent, Gent, Belgium
✉ jake.johnson@kuleuven.be

ABSTRACT

This supplementary information outlines the procedure to obtain the end of collection activities of the collected ^{225}Ac samples. Single-γ, $\gamma\gamma$, and α spectroscopy were performed on the samples in order to monitor the activity as a function of time. Details of the experimental setups as well as data analysis methods are included.

1 Spectroscopy of the ^{225}Ac Decay Chain

In order to measure the activity of the ^{225}Ac sources as a function of time, the rates of different decays shown in Fig. 1 were monitored. Two gamma-radiation detection setups were used. The first of these is the *lead castle setup*. Here a co-axial high purity germanium (HPGe) detector was used to obtain γ energy spectra in a very low background environment. Besides this, the *coincidence setup* measured $\gamma\gamma$ coincidence decay events with two HPGe detectors, allowing for efficiency-free activity determination. The final technique obtained α energy spectra with a Passivated Implanted Planar Silicon (PIPS) detector in the *alpha setup (ASET)*.

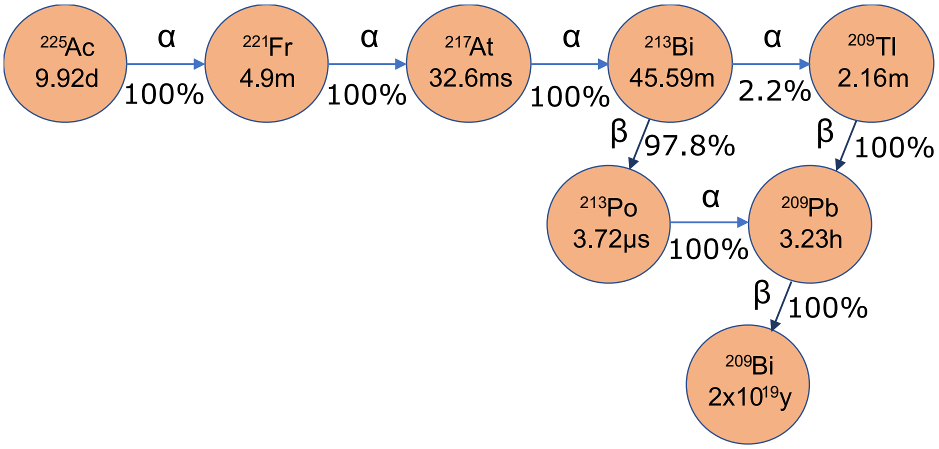

Figure 1. ^{225}Ac decay scheme. Half lives of the isotopes are indicated in the circles, and decay modes and branching ratios are indicated along the arrows.

2 Single γ-ray Spectroscopy in a Lead Castle

A lead castle, shown in Fig. 2, was used for measuring single γ-decay events in a low background environment. The most intense γ decay peaks in the ^{225}Ac decay chain are those at 218.12(2) keV and 440.45(1) keV which follow ^{221}Fr α-decay and

^{213}Bi β-decay respectively. The sources were placed in a glass vial at a distance of 25 cm from the detector. The γ photons were detected by a coaxial Canberra HPGe detector whose pre-amplified output signals were amplified and shaped by an ORTEC 572 amplifier (gain = 20, shaping time = 2μs), then read into an analog-to-digital converter (ADC) connected to a computer.

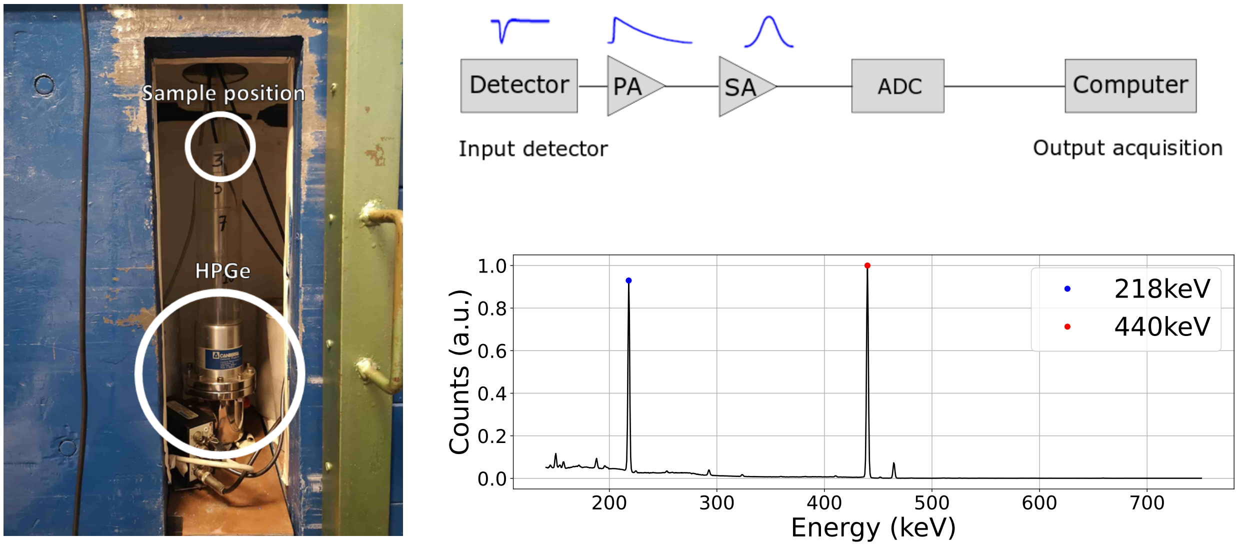

Figure 2. Image of the lead castle setup (left), electronic scheme for the setup (top right) and spectrum of one of the runs (bottom right).

Before the actual samples were measured, an energy and efficiency calibration were performed using three calibration sources (19.9 kBq ^{60}Co, 26.4 kBq ^{152}Eu and 27.6 kBq ^{133}Ba). The most intense peaks in the resulting spectra were fitted using a Gaussian function with a linear background. The mean channel of the Gaussian fit was used for the energy calibration. The obtained linear and quadratic energy calibration fits are shown together with their residues in Fig. 3 and the fit parameters are given in Table 1.

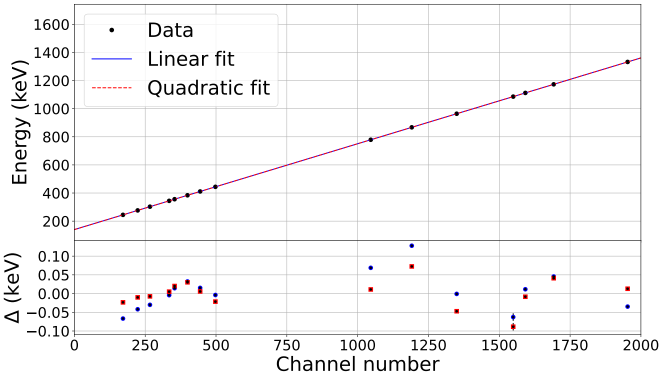

Figure 3. Fit for the Energy calibration (top) and its residues (bottom).

Table 1. Resulting fit parameters for the energy calibration.

Parameter	Linear	Quadratic
a	139.983(24)	139.897(29)
b	0.610611(21)	0.610885(74)
c	0	-1.30(35)
χ^2_{red}	0.00300009	0.00153670

The count rates in the most prominent γ peaks in the spectra of the calibration sources were compared to their γ activities to obtain an accurate estimate of the detection efficiency. These data points were fitted with the empirical fitting function in Equation (1), which is truncated at the third power of $\ln E$. The fit is shown in Fig. 4. The residues presented indicate an overestimation of the error. However, this is caused by the systematic effect induced by the uncertainty on the activity of the calibration sources. This error was propagated based on Monte Carlo sampling of the source activities from Gaussian

distributions 10^4 times (for each of the three sources). The efficiencies are calculated and fitted over the whole energy range for each triplet of activities, and the efficiency data is stored at an array of equally distributed energies. The systematic error band is then given by the values corresponding to $\pm 1\sigma$ at these points. Finally, the systematic error and the error obtained by propagating the fitting parameters are added in quadrature. The former of the two dominated the total error heavily. The obtained fit parameters are shown in Table 2. The efficiencies at 218 keV and 440 keV were determined to be 0.0979^{+34}_{-27} % and 0.0537^{+18}_{-15} % respectively.

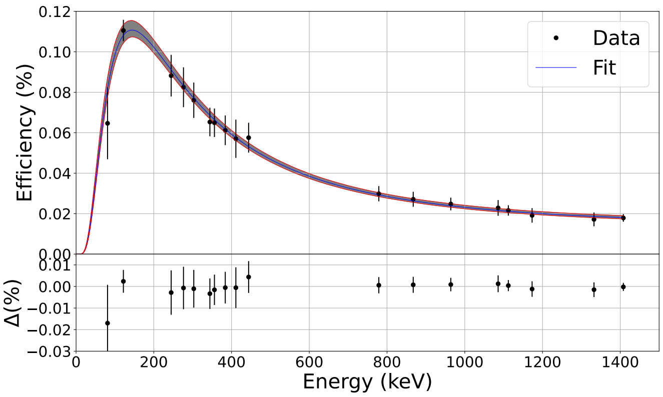

Figure 4. Fit for the Efficiency response function (top) and its residues (bottom).

$$f = Exp\left[a + b\ln(E) + c\ln(E)^2 + d\ln(E)^3\right] \quad (1)$$

Table 2. Resulting fit parameters for the efficiency response function.

Parameter	Value
a	-46.6(64)
b	22.8(34)
c	-3.78(57)
d	0.200(33)
χ^2_{red}	0.17421547

Dead-time and pile-up were investigated but determined to be negligible. Due to a slight deviation in the geometry, the measurements of the samples required a correction factor, calculated to be 1.0565(33). The mean time was used instead of the median time because the activity does not remain constant over the measurements period. Furthermore, Bateman correction factors were applied to convert the activity of a daughter isotope to that of ^{225}Ac. The obtained data points were fitted using an exponential with a fixed half-life of 9.92 days. The fit was then extrapolated to obtain the End Of Collection (EOC) activity. The obtained fits are shown in Fig. 5. A systematic shift in activities was observed between the different γ decays, which could have its origin in the efficiency response function, the source redistribution of ^{213}Bi compared to ^{221}Fr due to recoiling α decay daughters, or in the literature values for the intensities. A weighted average of these was taken to determine the EOC activity (shown in Table 3). For practical reasons, only the $^{225}\mathrm{Ac}^{\dagger}{}_{\mathrm{a}}$ and $^{225}\mathrm{Ac}^{\dagger}{}_{\mathrm{b}}$ sources were measured using the lead castle setup.

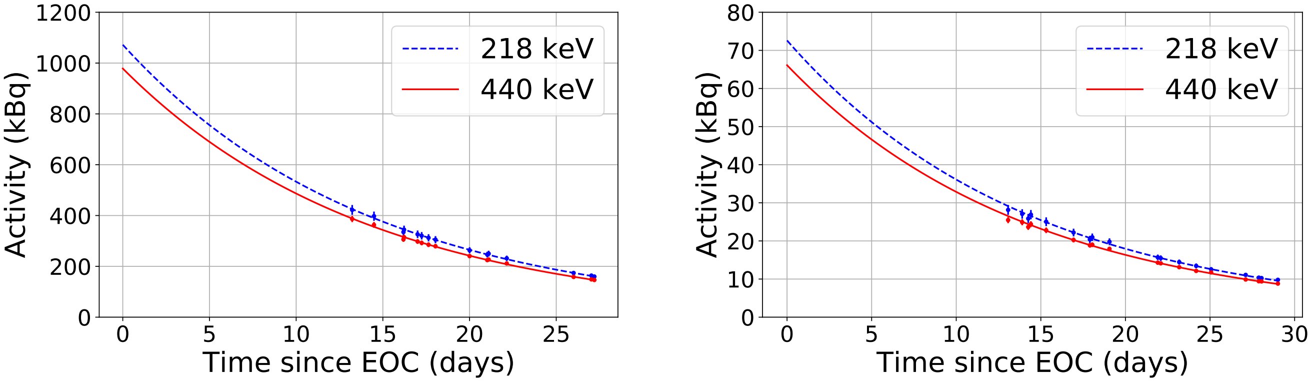

Figure 5. Exponential decay fits for the lead castle setup of sample $^{225}\mathrm{Ac}^{\dagger}{}_{\mathrm{a}}$ (left) and $^{225}\mathrm{Ac}^{\dagger}{}_{\mathrm{b}}$ (right).

Sample	Activity (kBq)	χ^2_{red}
$^{225}\mathrm{Ac}^{\dagger}{}_{\mathrm{a}}$	1009(34)	0.13892331
$^{225}\mathrm{Ac}^{\dagger}{}_{\mathrm{b}}$	68.2(23)	0.26407615

Table 3. Obtained EOC activities for the lead castle setup.

3 γ-γ Coincidence Measurements

Suppose two prompt γ-photons emitted in a cascade are measured by two separate detectors. In that case, it becomes possible to measure the activity of a sample without having an explicit dependence on the efficiency response of the detector. This is done by combining the count rates in a specific way, as shown in Equation (2).

$$A = \frac{N_1(E_1)N_2(E_2)}{N_{12}(E_1,E_2)} \frac{I_{12,\gamma}}{I_{1,\gamma}I_{2,\gamma}} \tag{2}$$

Here A is the activity of the source, $N_i(E)$ is the count rate of photons of energy E in detector i, $I_{i,\gamma}$ is the γ-intensity of the i'th peak, $I_{12,\gamma}$ is the intensity of the coincidences (which is not necessarily equal to $I_{1,\gamma}I_{2,\gamma}$) and $N_{12}(E_1,E_2)$ is the coincidence count rate for a photon with energy E_1 in detector 1 and E_2 in detector 2. It is possible to calculate $I_{12,\gamma}$ by using Equation 3. Here $I_{2,\gamma+ce}$ is the combined intensity of γ-decay and internal conversion, while α_2 is the internal conversion coefficient of the intermediate state.

$$\begin{aligned} I_{12,\gamma} = I_{1,\gamma}P(\gamma_2|\gamma_1) = I_{1,\gamma}\frac{I_{2,\gamma}}{I_{2,\gamma+ce}} = \frac{I_{1,\gamma}I_{2,\gamma}}{I_{2,\gamma}(1+\alpha_2)} \\ \implies \frac{I_{12,\gamma}}{I_{1,\gamma}I_{2,\gamma}} = \frac{1}{I_{2,\gamma}(1+\alpha_2)} \end{aligned} \tag{3}$$

The setup that was used is shown together with the electronic scheme in Fig. 6. The signal from either detector was first processed by its own pre-amplifier and red by a 14-bit digitizer (CAEN N674) and the data were collected event by event in a triggerless mode by the acquisition system (COMPASS). This allowed to reconstruct the analysis events via software post-analysis. The detectors were placed at a distance of 15 cm from the center of the source and at a relative angle of 90°. By doing this, the angular anisotropy would be equal to one for same-type multipolarity combinations. In the ^{225}Ac decay chain, two such coincidences have a sufficient intensity, namely an 807-293 keV pair and a 465-1567 keV pair following the decays of ^{213}Bi an ^{209}Tl respectively. However, as the transition multipolarities of the former are not known, and a systematic effect was found between the two, it was not used. There is likely a significant mixing present in at least one of its transitions. While the 465-1567 keV coincidences occur in nearly 96% of the ^{209}Tl-decays, the low branching towards this decay path makes it so that the coincidences are only present in about 2.06% of the ^{225}Ac-decays. Because of this, only the high activity ^{225}Ac samples, $^{225}\mathrm{Ac}^{\dagger}{}_{\mathrm{a}}$ and $^{225}\mathrm{Ac}^{\dagger}(\mathrm{ThO_2})$ could be measured using this setup.

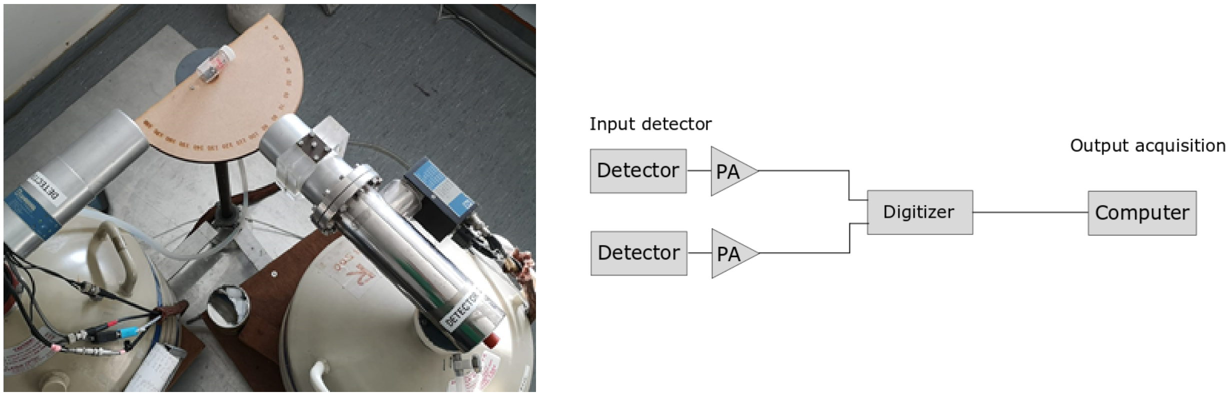

Figure 6. Image (left) and electronic scheme (right) of the coincidence setup.

The energy calibration was performed automatically in each run separately by finding the most intense peaks (218, 440 and 1567 keV) and applying linear regression. This value is typically accurate to less than 1 keV. For the direct count rates, the background of the spectrum over a given measurement was subtracted using a specialized ROOT function[1]. The spectrum is shown in Fig. 7. The red line indicates the estimated background, which provides a spectrum of Gaussian peaks once subtracted. The bin integral was taken within 5 channels before and after the peak position to obtain the single count rates. Furthermore, the coincidence window was set to be ±200 ns, corresponding to the detector timing resolution shown in Fig. 7.

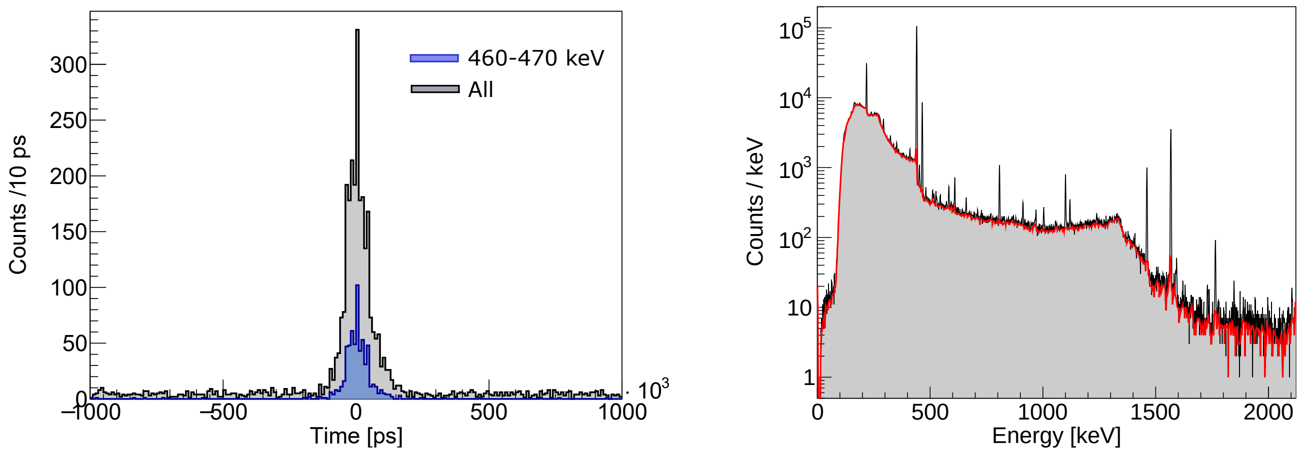

Figure 7. Coincidences as a function of time difference between the two detectors (left) and spectrum with estimated background for the coincidence setup (right).

After setting an energy gate around the energy of the *trigger photon*, the energy of the coincidence particles can be studied. These single energy-gated spectra are given in Fig. 8. The left and right graphs show the spectra gated on 465 and 1567 keV photons respectively. For the first, true coincidences appear at 117 and 1567 keV. The single count rates of the former could not be obtained to a sufficient precision, so this coincidence was omitted from the analysis. Furthermore, random coincidences occur most significantly for γ photons of energies 218 keV and 440 keV. Besides that, a Compton continuum of the 1567 keV peak is present in the spectrum. Finally, the trigger photon can be an already Compton scattered 1567 keV particle. This can be interpreted as a *false trigger*. Because of this, a minor true coincidence peak is present at the 465 keV trigger energy. A less complex spectrum is obtained with a 1567 keV trigger photon. True coincidences are present at 117 and 465 keV, while the random coincidences are less prominent. Finally, there is a Compton continuum originating mainly from the 465 keV photon.

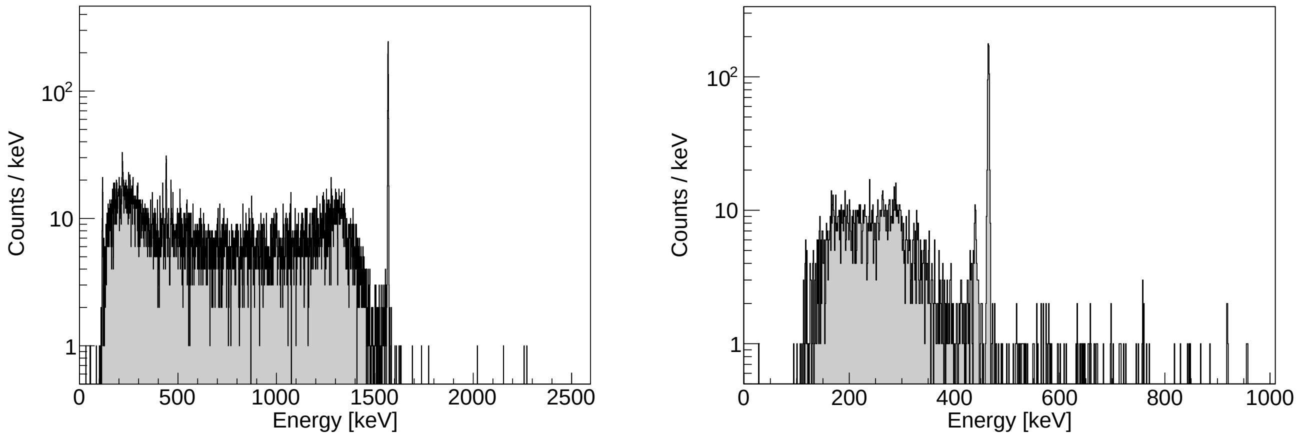

Figure 8. Energies in coincidence with a 465 keV (left) and 1567 keV (right) trigger photon.

Due to switching between setups and refilling liquid nitrogen for the cooling of the detectors, the measurements were made in several batches. As the file size was limited in COMPASS to 50 MB, each batch consisted of several linked files in a chain. To obtain multiple data points per chain, they were split such that each data point had several coincidence counts. The rate of data points can be kept similar over different batches by changing the number of entries within a data point following a scaling given by Equation (4). Here N_f is the number of entries per data point chosen for the last point, λ is the decay constant of ^{225}Ac, and t_f and t_i are the mean times of the final batch and data point i respectively. As both the rate of events and the number of entries within a data point are exponentially decreasing, the number of data points over a given period remains the same (when neglecting background).

$$N_i = N_f e^{\lambda(t_f - t_i)} \tag{4}$$

Due to the nature of the measurement technique, dead-time and pile-up do not affect the obtained activity. However, a Bateman correction factor must still be applied to convert the ^{209}Tl activity to the ^{225}Ac activity. The obtained exponential fits are shown in Fig. 9 and the EOC activities are shown in Table 4.

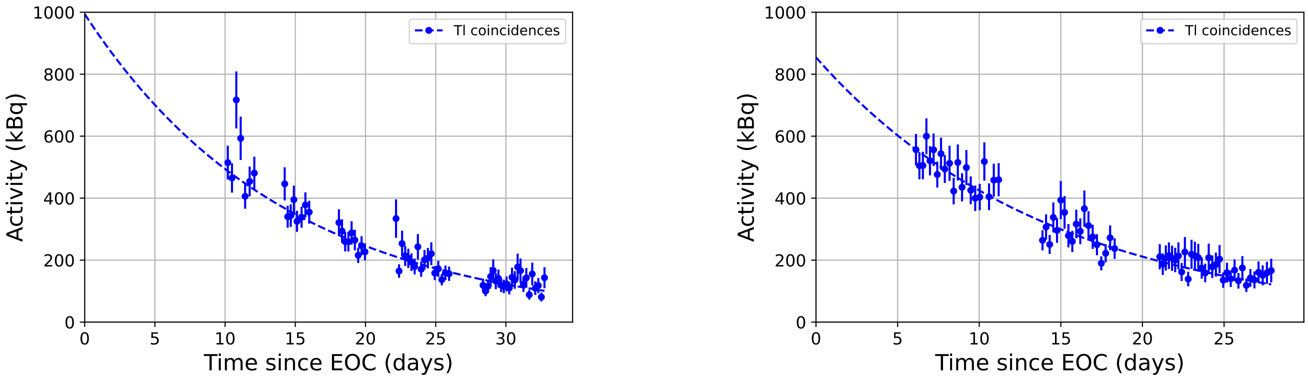

Figure 9. Exponential decay fits for the coincidence setup of sample $^{225}\text{Ac}^{\dagger}{}_{\text{a}}$ (left) and $^{225}\text{Ac}^{\dagger}(\text{ThO}_2)$ (right).

Table 4. Obtained EOC activities for the coincidence setup.

Sample	Activity (kBq)	χ^2_{red}
$^{225}\text{Ac}^{\dagger}{}_{\text{a}}$	996(19)	1.01960902
$^{225}\text{Ac}^{\dagger}(\text{ThO}_2)$	857(14)	0.83041686

4 Alpha spectroscopy

Alpha decay spectroscopy was performed with a Canberra partially depleted 300mm^2 active area PIPS detector with 150μm thickness. The sample foil was manipulated with tweezers and mounted at a fixed distance of 20.3(2)mm from the PIPS detector. The measurements were performed in vacuum at pressures less than 10^{-5}mbar. Alpha energy spectra were obtained from this measurement campaign by collecting the data from the pre-amplifier directly into a 14-bit digitzer (CAEN N674), recorded in a triggerless mode (COMPASS), and analysed to determine the activity of ^{225}Ac at the mean time of each acquired spectrum.

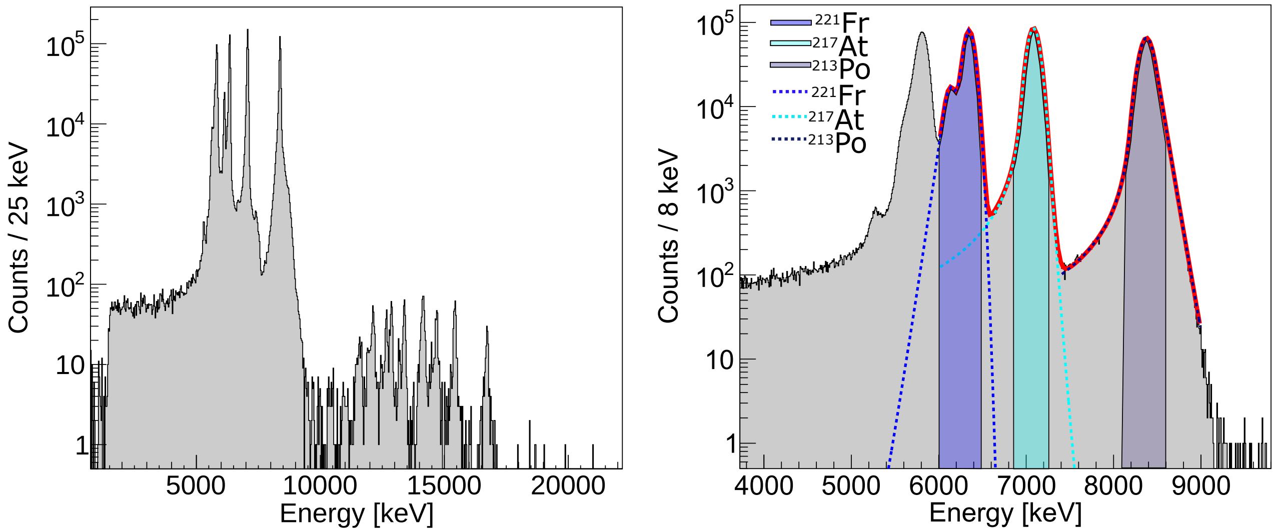

Figure 10. Left: a typical α decay energy spectrum of the $^{225}\mathrm{Ac}^{\dagger}{}_{\mathrm{a}}$ source. Peaks in the energy region greater than 10000 keV are due to $\alpha - \alpha$ summing and were not counted in the analysis as their relative contribution to the total count rate was lower than 1%. Right: A typical α decay energy spectrum of $^{225}\mathrm{Ac}^{\dagger}(\mathrm{ThO_2})$ used for calculating the correction factors $c_{pu}(X)$. The shaded areas are integrated counts in the energy range used to derive count rate, while the ratio of the area under the dashed lines to the shaded area constitutes $c_{pu}(X)$ for each isotope considered.

Firstly, the count rates of the $^{213}\mathrm{Po}$, $^{217}\mathrm{At}$ and $^{221}\mathrm{Fr}$ α decay peaks were determined by integrating the differential energy spectra in the energy windows 8100-8600, 6850-7250 and 6000-6500 keV respectively and dividing by the measurement duration to obtain a detected count rate. The $^{225}\mathrm{Ac}$ and $^{213}\mathrm{Bi}$ peaks could not be resolved independently and were not used in the analysis. In order to convert the count rate from nuclide X to the activity of $^{225}\mathrm{Ac}$ Equation. 5 was used.

$$A^{(^{225}Ac)}(t) = \frac{r^{(X)}(t)c_{pu}(t)}{b_X \varepsilon_{geo}^{(X)} \varepsilon_{int}(1 - R(t)\tau)} \tag{5}$$

Here $r^{(X)}(t)$ is the count rate of α decays from nuclide X, $c_{pu}(t)$ is a correction factor accounting for the α decay counts outside of the prescribed energy windows, b_X is the intensity of α decays with respect to an $^{225}\mathrm{Ac}$ decay in secular equilibrium, $\varepsilon_{geo}^{(X)}$ is the geometric efficiency of nuclide X, ε_{int} is the intrinsic efficiency of the detector (=100%), R(t) is the registered count rate of the detector and τ is the detector dead-time. The correction factor c_{pu} is implicitly time-dependent through its dependence on the source activity. Furthermore, the geometric efficiency is different for each nuclide due to source redistribution following the recoil induced by α decay. Finally, the *live-time* fraction is given by $1 - R(t)\tau$.

The factor $c_{pu}(t)$ was calculated by fitting 'by hand' a series of spectra along the measurement campaign, using an adapted Gaussian function with both high and low energy Crystal Ball-like tails[2,3]. This accounts for a combination of straggling, summing, detector degradation and pulse pileup effects for each principal α decay peak. Fits were performed using this function for each of the Po, At and Fr energy regions. The ratio of the area of the fitted functions to the integrated counts in the aforementioned energy ranges for each spectrum gave the isotope- and time-dependent correction factors.

The dead-time τ was calculated employing the 'decaying source method' using the actinium decay itself. More details of this method can be found in[4]. The dead-time was determined to be 1.49 μs and 1.04 μs during decay spectroscopy of the $^{225}\mathrm{Ac}^{\dagger}{}_{\mathrm{a}}$ source and $^{225}\mathrm{Ac}^{\dagger}(\mathrm{ThO_2})$ source respectively. The dead-time could not be calculated for measurements of the $^{225}\mathrm{Ac}^{\dagger}{}_{\mathrm{b}}$ source due to the low source activity.

Finally, the factor $\varepsilon_{geo}^{(X)}$ was calculated numerically based on layered SRIM simulations[5]. This was especially necessary as the $^{225}\mathrm{Ac}$ daughters' recoil energy were greater than the implantation energy, so with each decay the distribution of the daughter nuclide position covered the foil, the detector, and elsewhere in the system. The obtained geometric efficiencies are given in Table 5. The relative uncertainty was obtained by adding the statistical uncertainty (Poisson statistics) and an estimated systematic error in quadrature.

Table 5. Geometric efficiencies at 20.3(2) cm source-detector distance for selected isotopes obtained via layered SRIM simulations. The resulting EOC activities from each isotope are given for each source.

Isotope	Geometric efficiency (%)	$^{225}Ac^{\dagger}{}_{a}$ activity (kBq)	$^{225}Ac^{\dagger}{}_{b}$ activity (kBq)	$^{225}Ac^{\dagger}(ThO_2)$ activity (kBq)
^{221}Fr	4.820(70)[100]	961(24)	62.5(16)	1030(27)
^{217}At	4.963(71)[100]	904(22)	60.1(15)	970 (25)
^{213}Po	4.568(68)[100]	949(24)	61.7(16)	1050 (29)
Weighted mean	/	936(14)	61.4(9)	1013(16)

5 End of collection activity

The final end of collection activity of ^{225}Ac for each sample was determined as a weighted average of the those calculated from each of the three setups described above. The weights are given by the inverse square of the uncertainty of the calculated activity. The ^{225}Ac activity from each setup was calculated as the weighted mean of the daughter isotopes whose activities were determined, with the appropriate factor accounting for secular equilibrium. For the lead castle setup this constituted ^{221}Fr and ^{213}Bi. For the coincidences setup ^{209}Tl, only. For the ASET, ^{221}Fr, ^{217}At and ^{213}Po were used. Table 6 shows the ^{225}Ac activities calculated from each setup as well as their uncertainties. These are the values that were used in the main text to determine the efficiency.

Table 6. The end of collection ^{225}Ac activities of each collected sample from each of the three setups.

Sample	γ activity (kBq)	$\gamma\gamma$ activity (kBq)	α activity (kBq)	Sample activity (kBq)	Collection Efficiency (%)
$^{225}Ac^{\dagger}{}_{a}$	1009(34)	996(19)	936(14)	962(22)	9.5(2)
$^{225}Ac^{\dagger}{}_{b}$	68.2(23)	/	61.4(9)	62.3(2.3)	0.63(2)
$^{225}Ac^{\dagger}(ThO_2)$	/	857(14)	1013(16)	925(77)	9.9(8)

It is notable that the calculated activities between the α setup and the other two setups do not fully agree within the uncertainties provided. This is most probably due to a systematic effect in the geometric efficiency of the α setup. The source re-distribution of daughter isotopes in the decay chain was modelled using SRIM, which does not account for all possible processes including self-sputtering of the source, which would lead to a systematic shift in the calculated geometric efficiency. Furthermore, the mounted PIPS detector was not perfectly aligned parallel to the sample foil, which would also induce a systematic shift in the detection efficiency.

References

1. Antcheva, I. *et al.* ROOT–A C++ framework for petabyte data storage, statistical analysis and visualization. *Comput. Phys. Commun.* **182**, 1384–1385 (2011).

2. Skwarnicki, T. *A Study of the Radiative Cascade Transitions between the Upsilon-Prime and Upsilon Resonances*. Ph.D. thesis, Institute of Nuclear Physics, Cracow, Poland (1986). DESY-F31-86-02.

3. Gaiser, J. *Charmonium Spectroscopy from Radiative Decays of the J/ψ and ψ'*. Ph.D. thesis, Stanford University, Stanford, California, USA (1982). SLAC-0255.

4. Knoll, G. F. *Radiation detection and measurement* (John Wiley & Sons, 2010).

5. Ziegler, J. F., Ziegler, M. D. & Biersack, J. P. SRIM–the stopping and range of ions in matter (2010). *Nucl. Instruments Methods Phys. Res. Sect. B: Beam Interactions with Mater. Atoms* **268**, 1818–1823 (2010).
