# Supplementary figures and images for: Resonant laser ionization and mass separation of 225Ac
Source: Sci Rep. 2023 Jan 24;13:1347. doi: 10.1038/s41598-023-28299-4 (PMC9873802; doi:10.1038/s41598-023-28299-4)

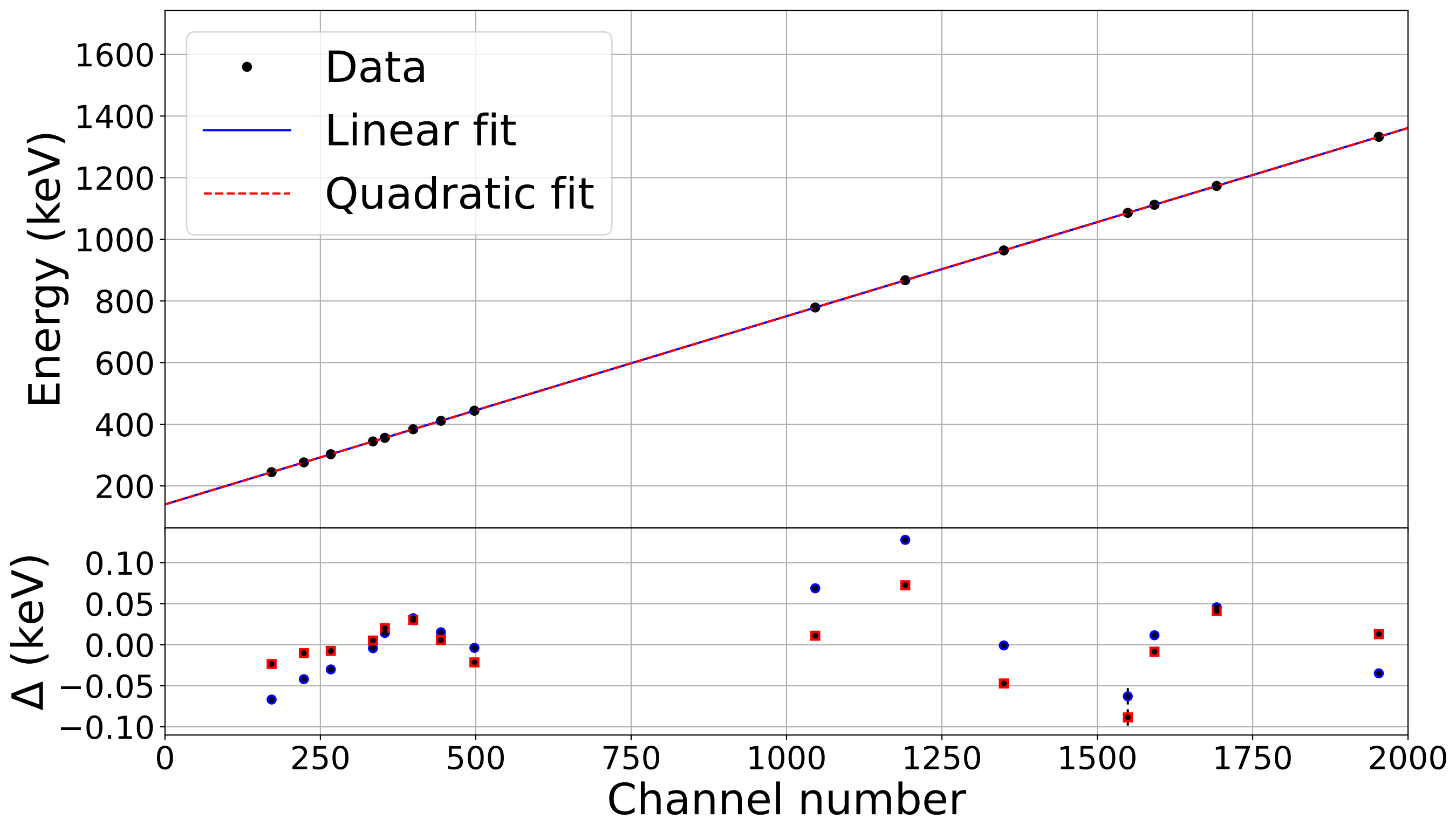

Supplement: Supplementary file 1 — Supplementary Information. [file 41598_2023_28299_MOESM1_ESM.zip › Images/calibration.pdf]

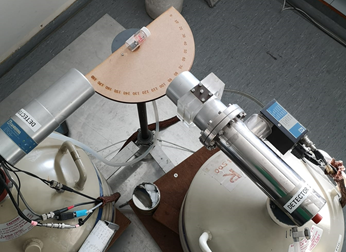

Supplement: Supplementary file 1 — Supplementary Information. [file 41598_2023_28299_MOESM1_ESM.zip › Images/coincidence_setup.png]

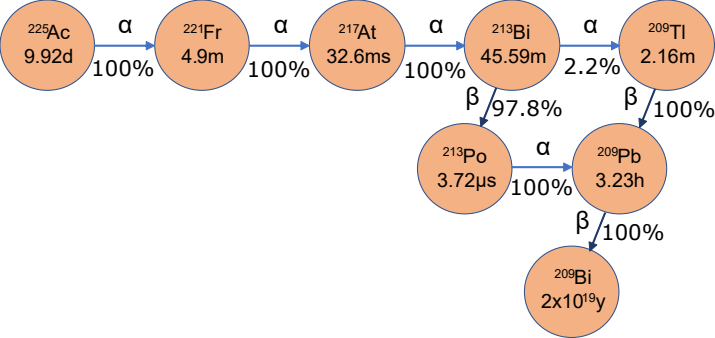

Supplement: Supplementary file 1 — Supplementary Information. [file 41598_2023_28299_MOESM1_ESM.zip › Images/decay_schema_branchings.pdf]

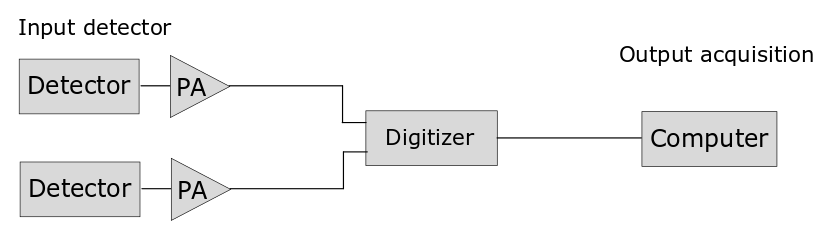

Supplement: Supplementary file 1 — Supplementary Information. [file 41598_2023_28299_MOESM1_ESM.zip › Images/electronic_scheme_coinc.png]

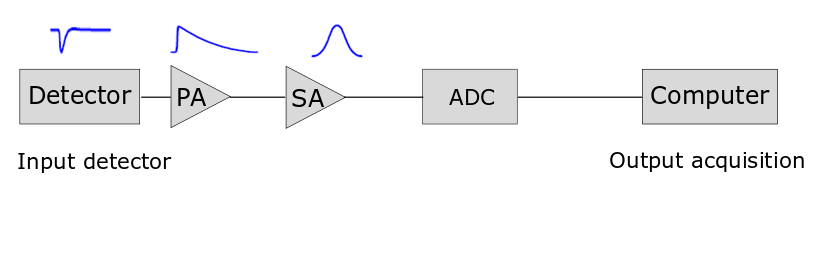

Supplement: Supplementary file 1 — Supplementary Information. [file 41598_2023_28299_MOESM1_ESM.zip › Images/electronic_scheme_Pb.png]

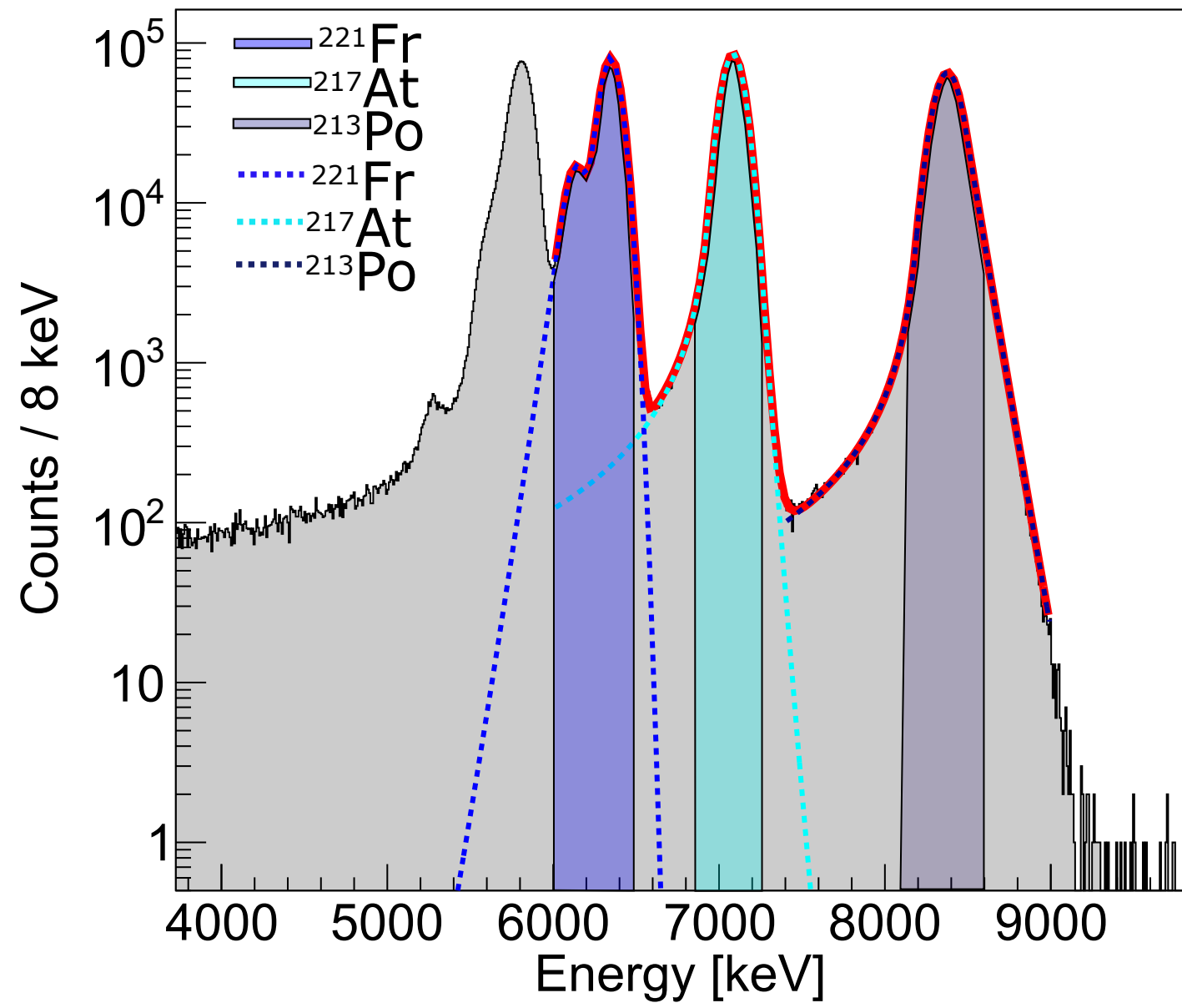

Supplement: Supplementary file 1 — Supplementary Information. [file 41598_2023_28299_MOESM1_ESM.zip › Images/fitted_m120_peaks_correction_factor_regions.pdf]

Activity (kBq)

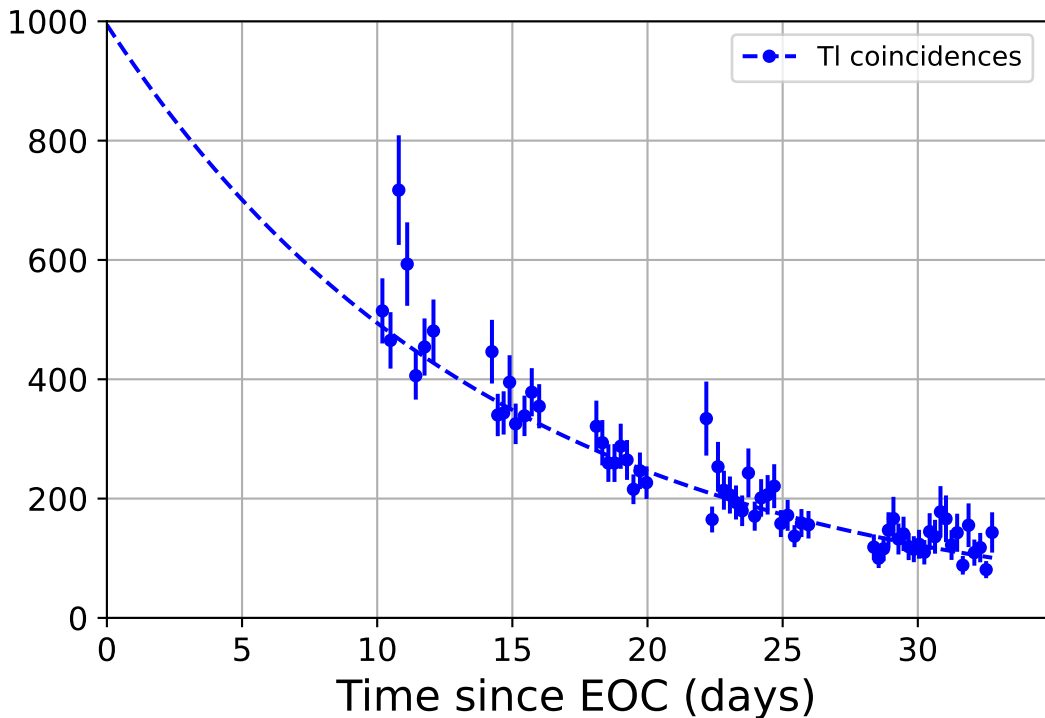

Supplement: Supplementary file 1 — Supplementary Information. [file 41598_2023_28299_MOESM1_ESM.zip › Images/M108_activity_coincidences.pdf]

Activity (kBq)

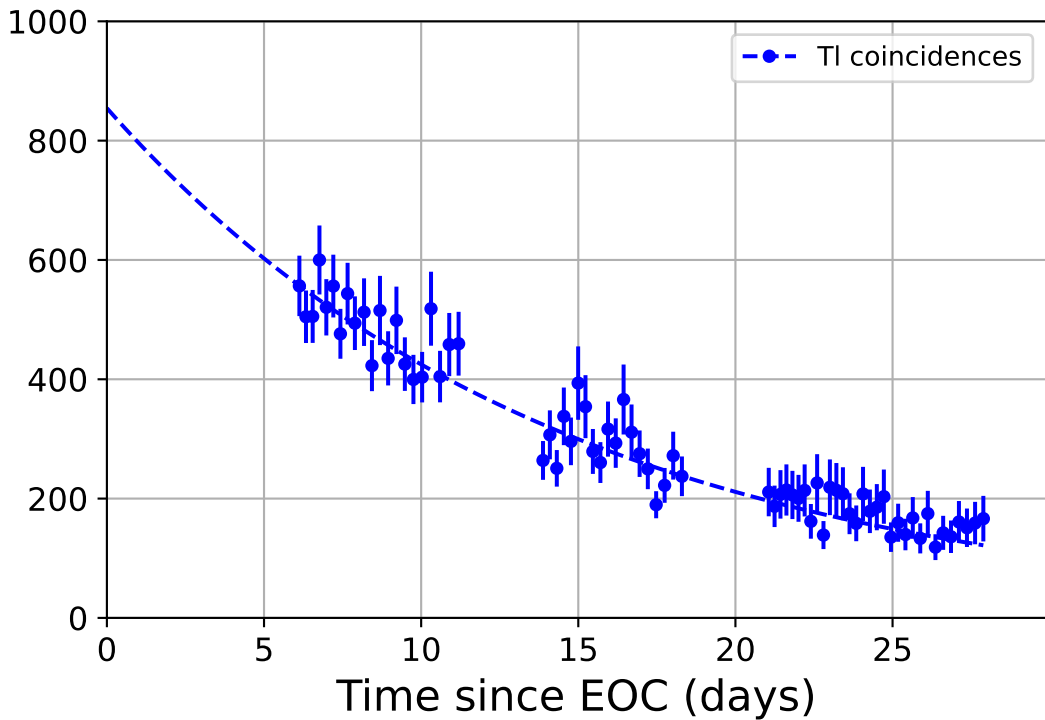

Supplement: Supplementary file 1 — Supplementary Information. [file 41598_2023_28299_MOESM1_ESM.zip › Images/M120_activity_coincidences.pdf]

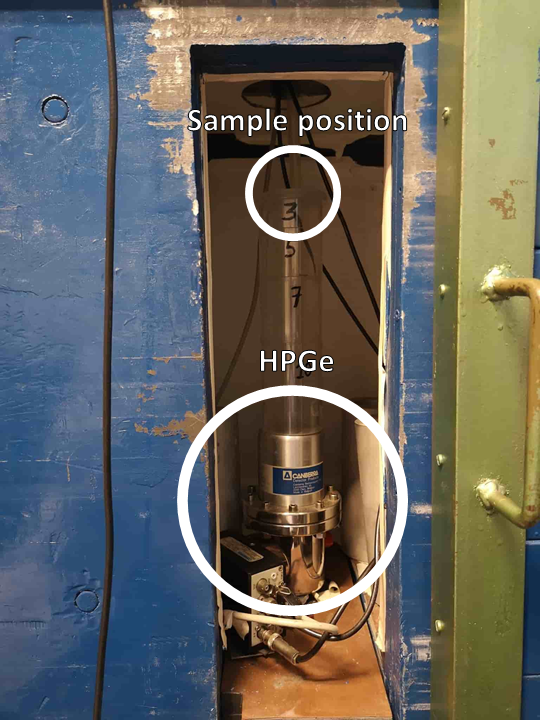

Supplement: Supplementary file 1 — Supplementary Information. [file 41598_2023_28299_MOESM1_ESM.zip › Images/Pb_castle.png]

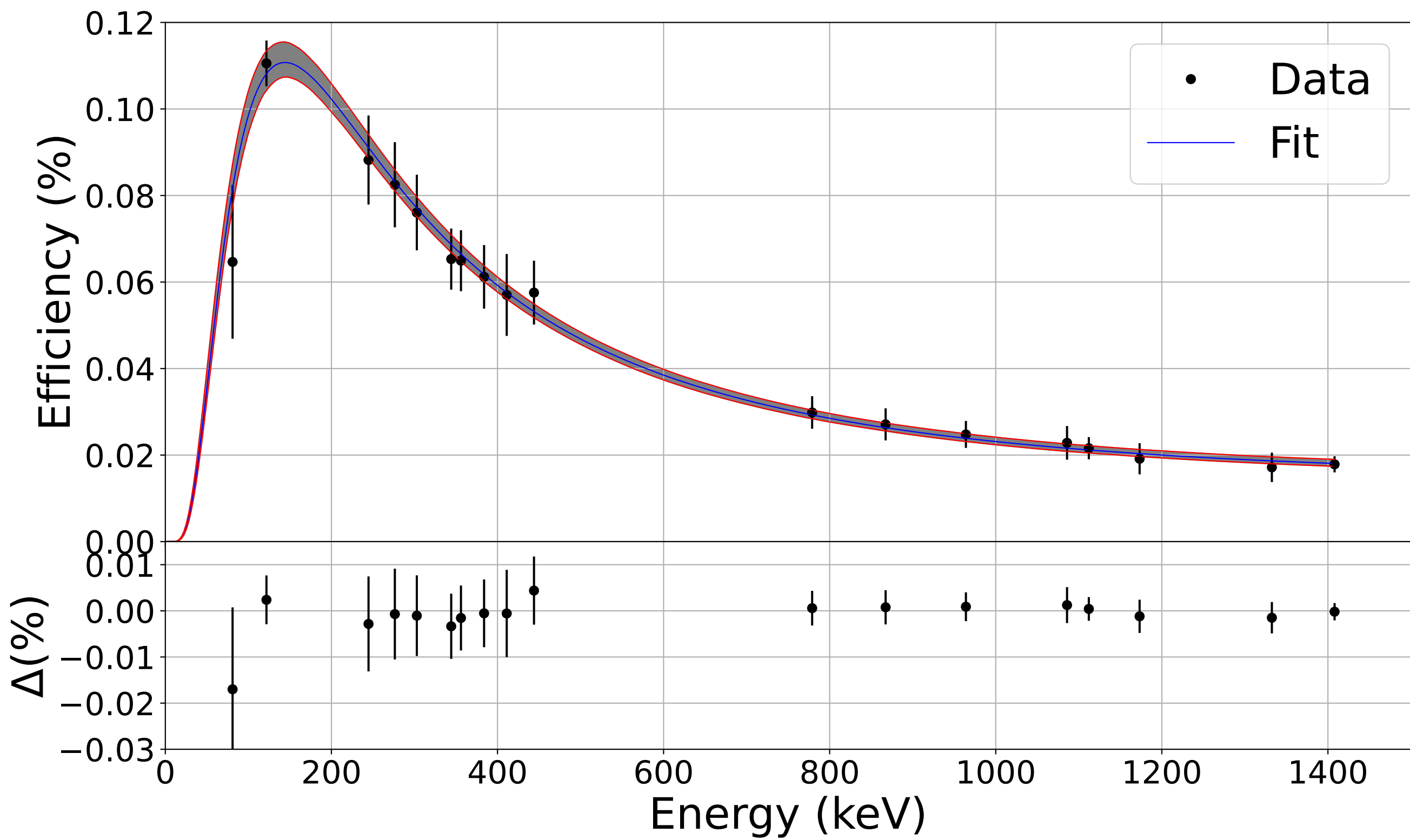

Supplement: Supplementary file 1 — Supplementary Information. [file 41598_2023_28299_MOESM1_ESM.zip › Images/pb_efficiency.pdf]

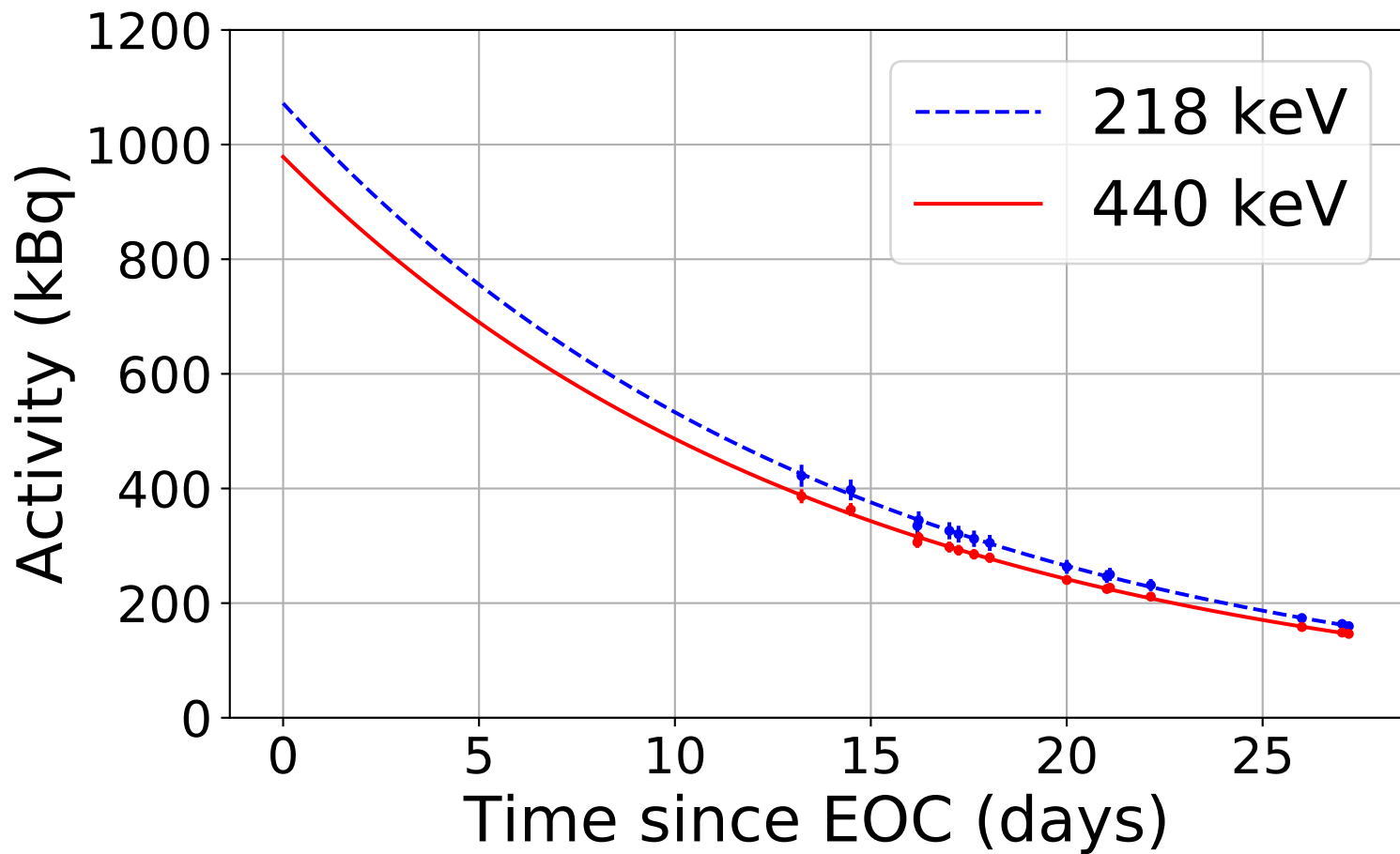

Supplement: Supplementary file 1 — Supplementary Information. [file 41598_2023_28299_MOESM1_ESM.zip › Images/Pb_M108.pdf]

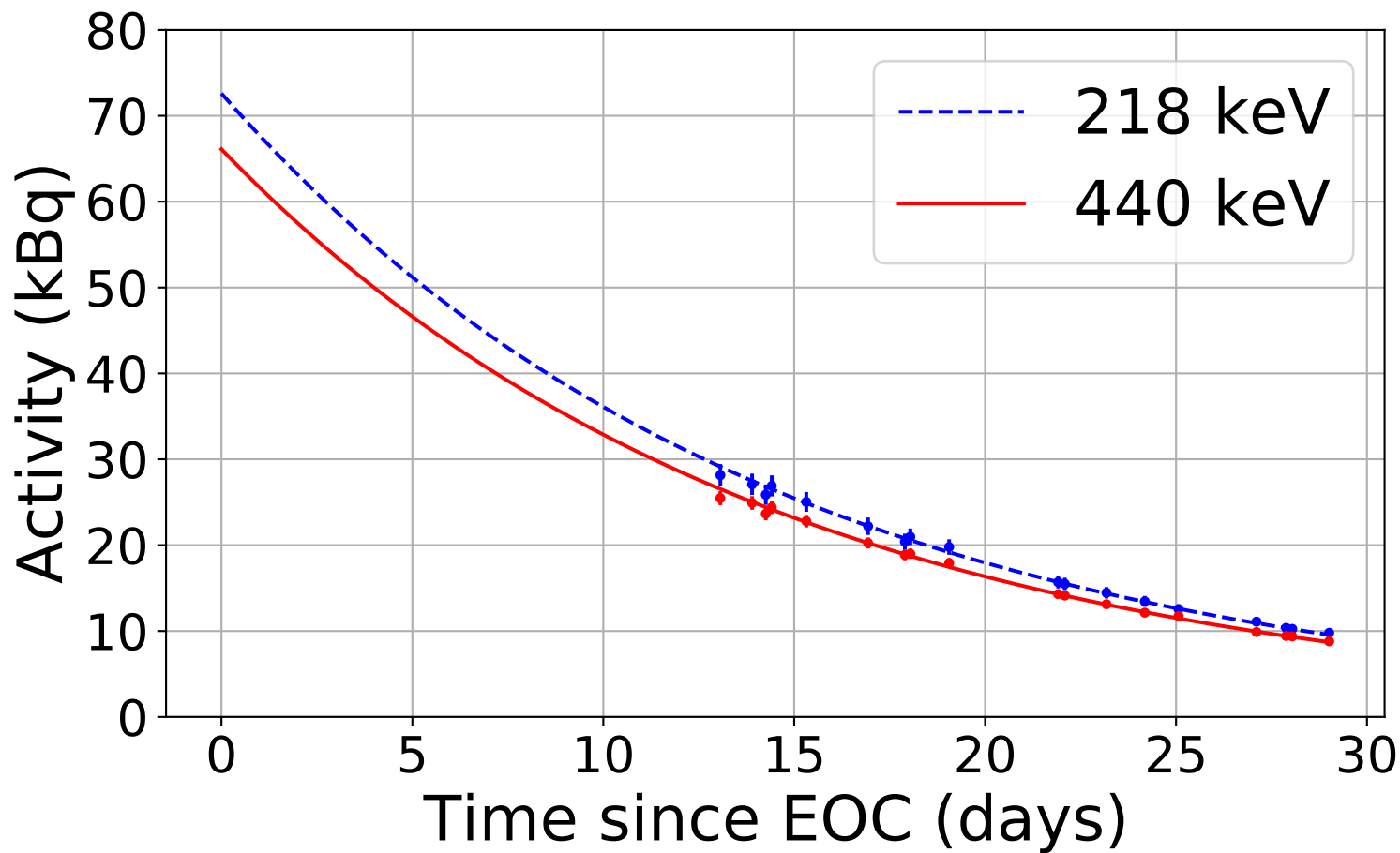

Supplement: Supplementary file 1 — Supplementary Information. [file 41598_2023_28299_MOESM1_ESM.zip › Images/Pb_M118.pdf]

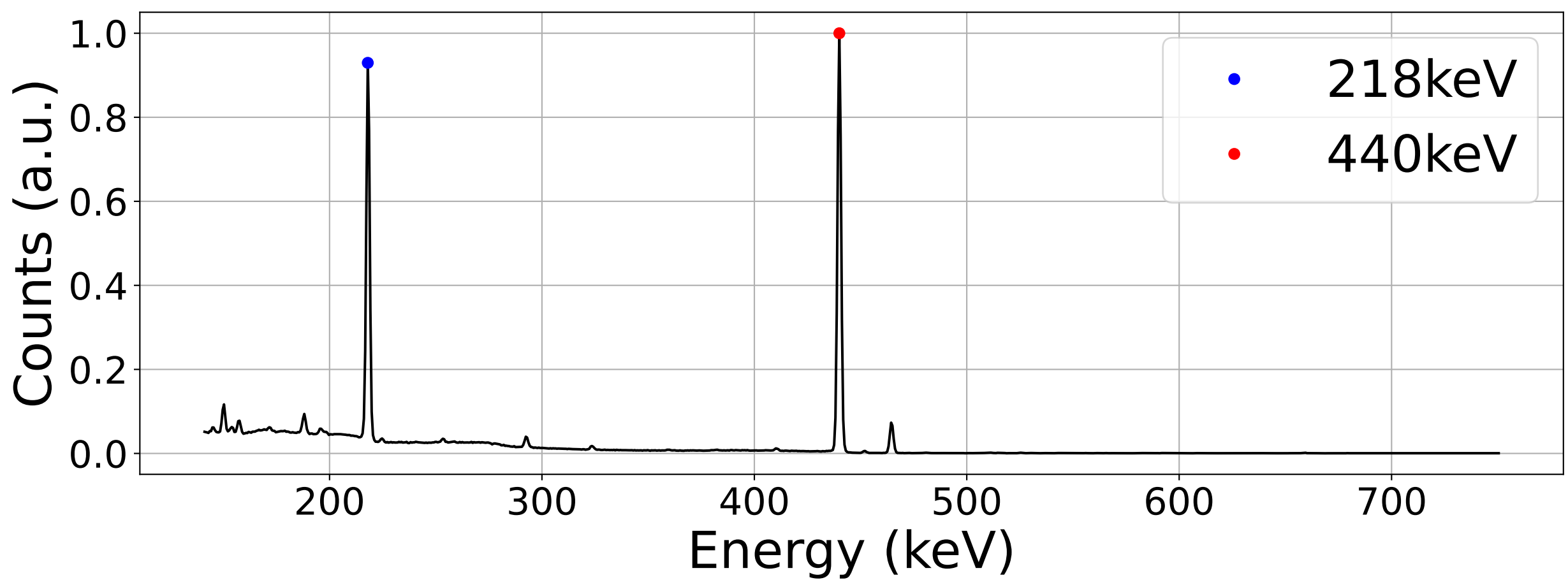

Supplement: Supplementary file 1 — Supplementary Information. [file 41598_2023_28299_MOESM1_ESM.zip › Images/Pb_spectrum.pdf]

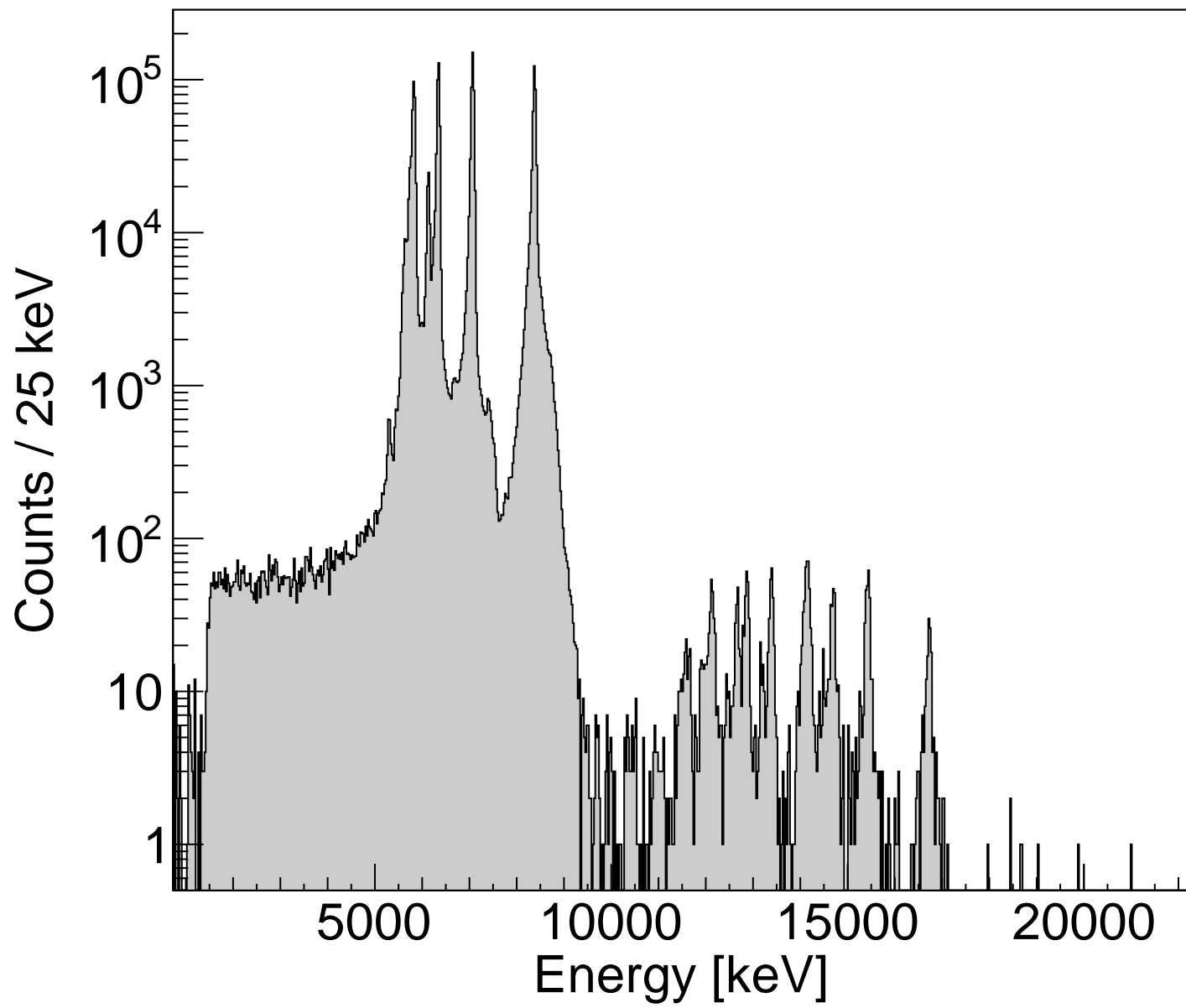

Supplement: Supplementary file 1 — Supplementary Information. [file 41598_2023_28299_MOESM1_ESM.zip › Images/publication_supp_m108_alpha_spectrum.pdf]

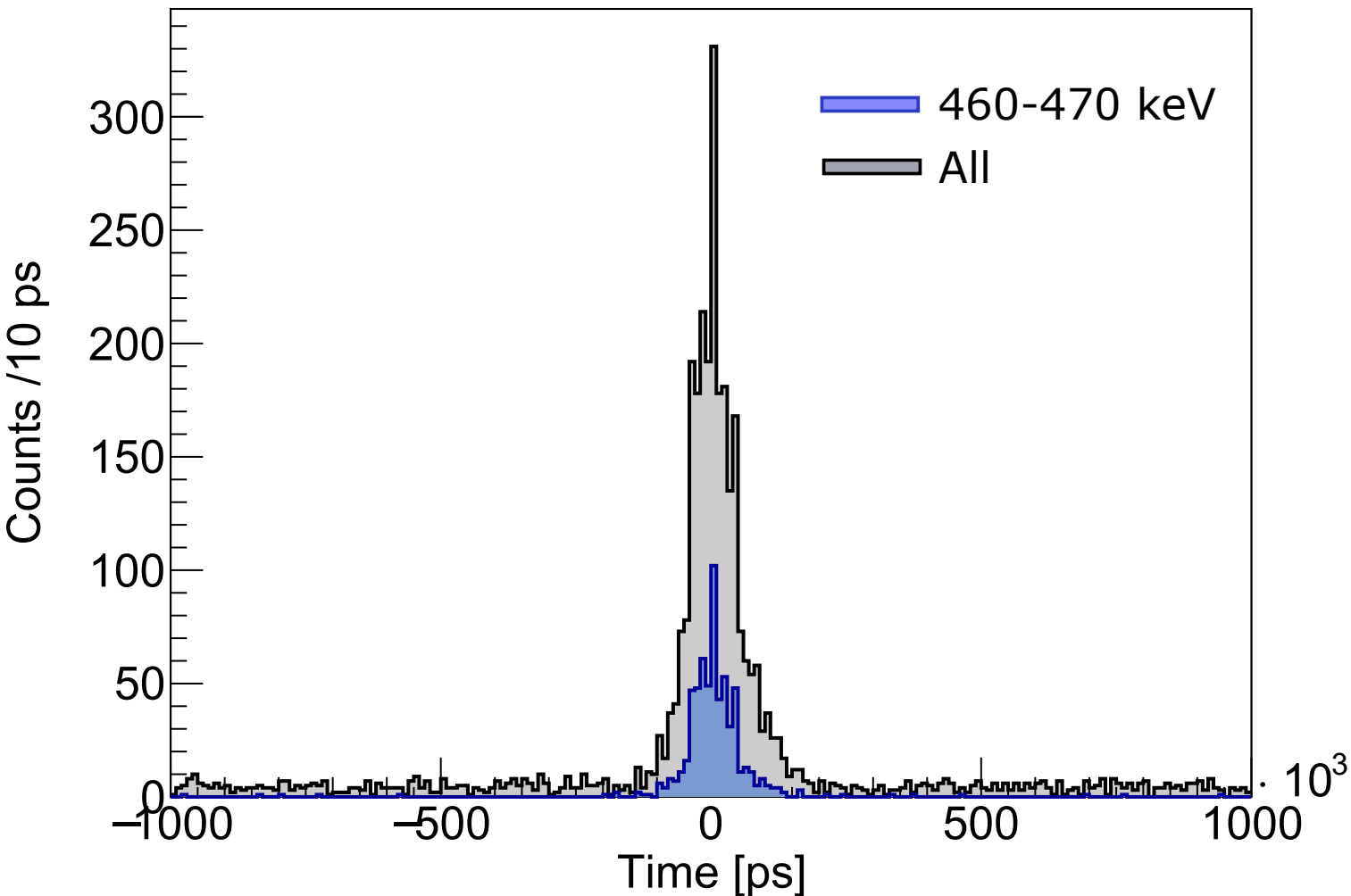

Supplement: Supplementary file 1 — Supplementary Information. [file 41598_2023_28299_MOESM1_ESM.zip › Images/supp_mat_200ns_window_IS.pdf]

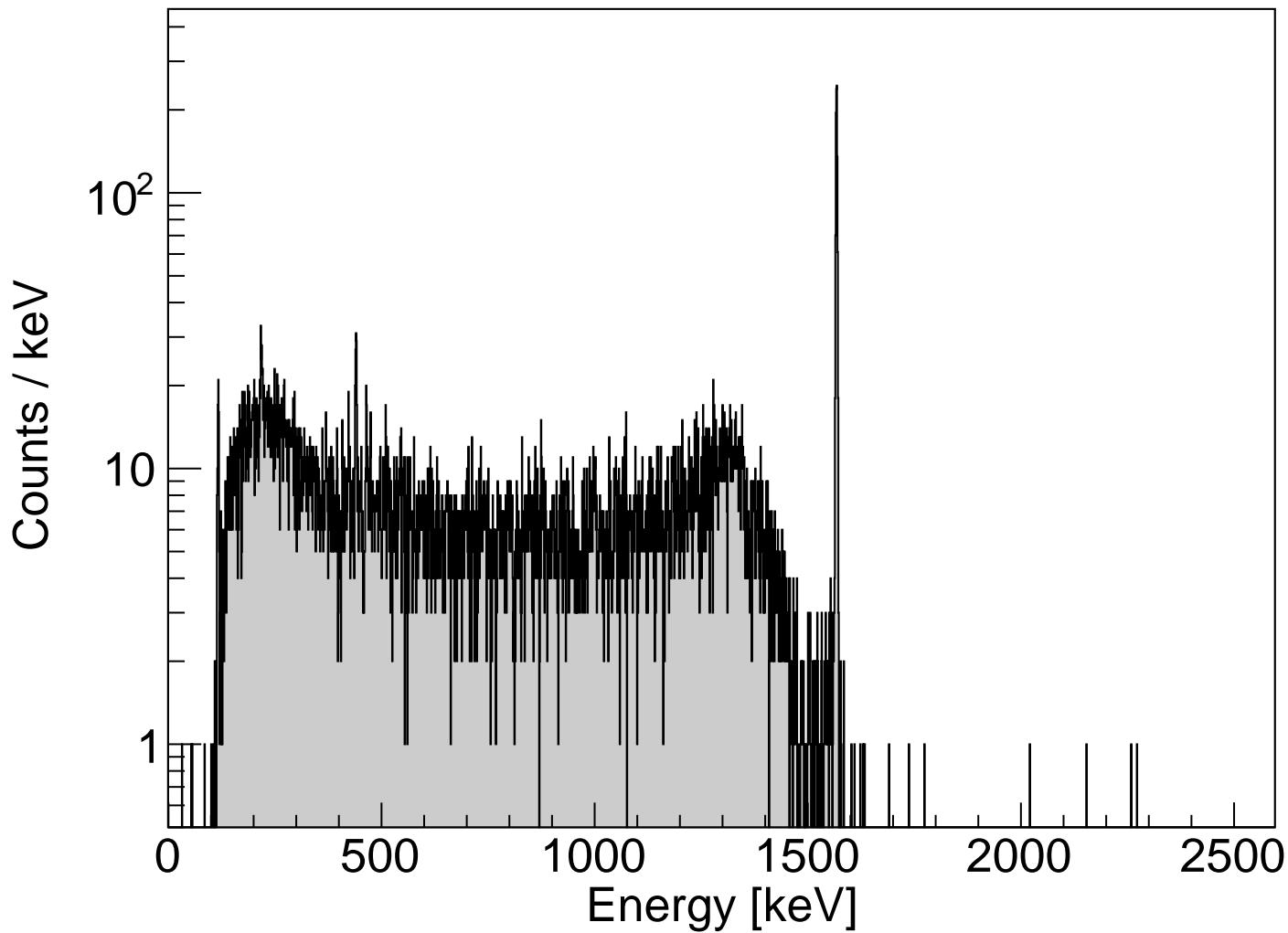

Supplement: Supplementary file 1 — Supplementary Information. [file 41598_2023_28299_MOESM1_ESM.zip › Images/supp_mat_465_coincidence.pdf]

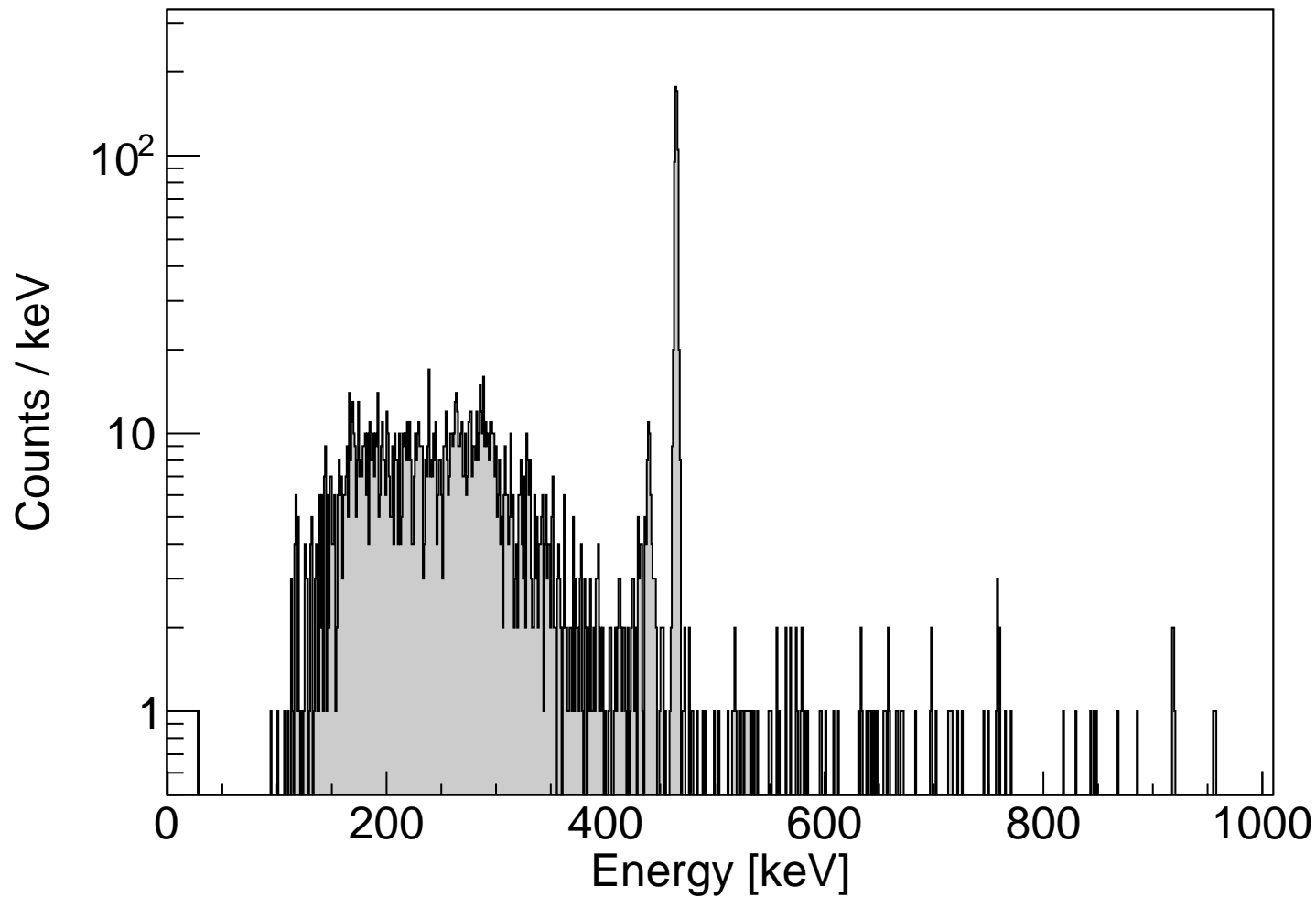

Supplement: Supplementary file 1 — Supplementary Information. [file 41598_2023_28299_MOESM1_ESM.zip › Images/supp_mat_1567_coincidence.pdf]

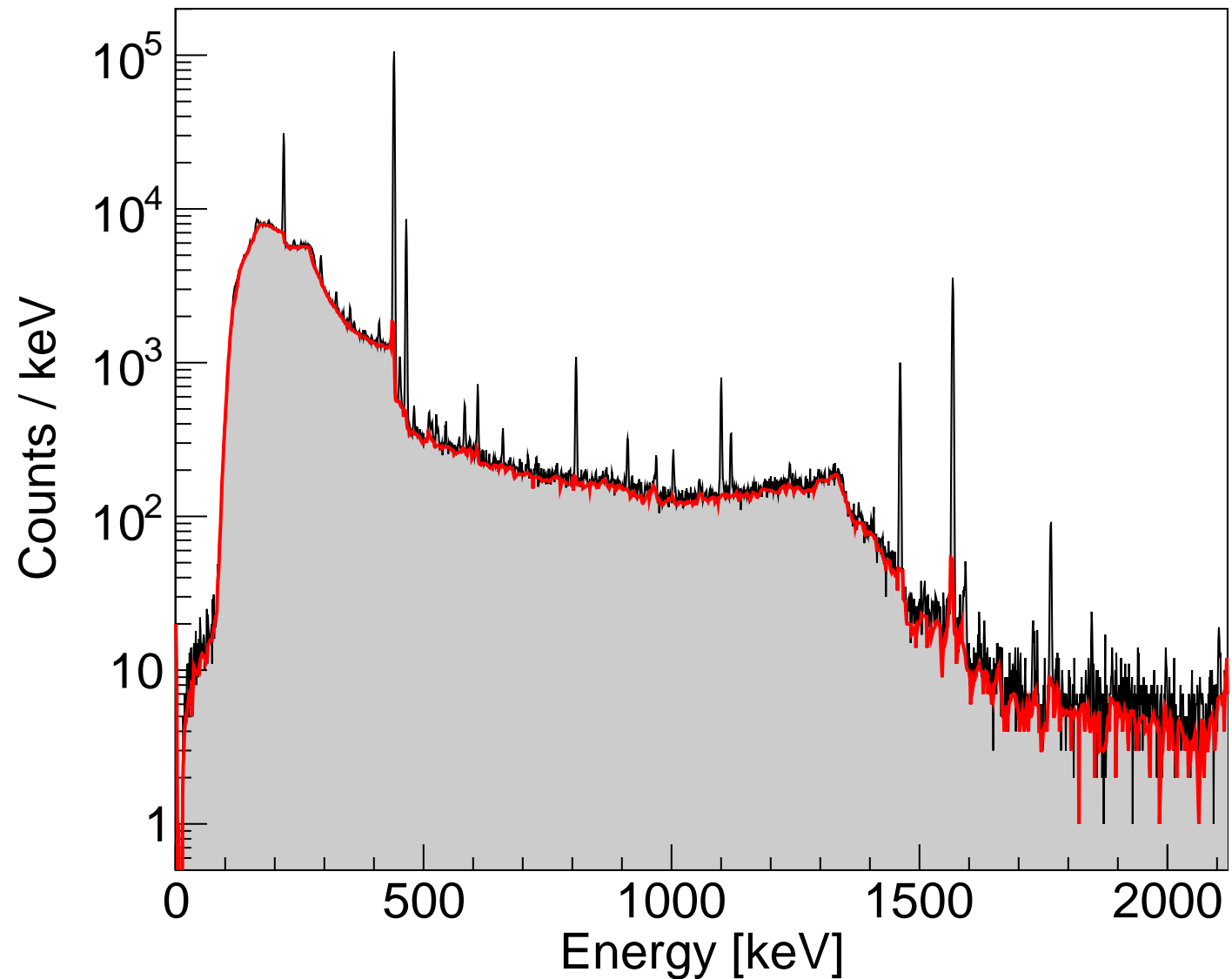

Supplement: Supplementary file 1 — Supplementary Information. [file 41598_2023_28299_MOESM1_ESM.zip › Images/supp_mat_spectrum_with_background.pdf]
